# Identification of a *RAI1*-associated disease network through integration of exome sequencing, transcriptomics, and 3D genomics

**DOI:** 10.1186/s13073-016-0359-z

**Published:** 2016-11-01

**Authors:** Maria Nicla Loviglio, Christine R. Beck, Janson J. White, Marion Leleu, Tamar Harel, Nicolas Guex, Anne Niknejad, Weimin Bi, Edward S. Chen, Isaac Crespo, Jiong Yan, Wu-Lin Charng, Shen Gu, Ping Fang, Zeynep Coban-Akdemir, Chad A. Shaw, Shalini N. Jhangiani, Donna M. Muzny, Richard A. Gibbs, Jacques Rougemont, Ioannis Xenarios, James R. Lupski, Alexandre Reymond

**Affiliations:** 1 Center for Integrative Genomics, University of Lausanne, 1015 Lausanne, Switzerland; 2 Department of Molecular and Human Genetics, Baylor College of Medicine, Houston, TX 77030 USA; 3 School of Life Sciences, EPFL (Ecole Polytechnique Fédérale de Lausanne), 1015 Lausanne, Switzerland; 4 Swiss Institute of Bioinformatics (SIB), 1015 Lausanne, Switzerland; 5 Human Genome Sequencing Center, Baylor College of Medicine, Houston, TX 77030 USA; 6 Department of Pediatrics, Baylor College of Medicine, Houston, TX 77030 USA; 7 Texas Children’s Hospital, Houston, TX 77030 USA; 8 Laboratory Medicine Program, UHN, Department of Laboratory Medicine and Pathobiology, University of Toronto, Toronto, ON M5G 2C4 Canada; 9 Present address: WuXiNextCODE, 101Main Street, Cambridge, MA 02142 USA

**Keywords:** Diagnostic, Intellectual disability, Chromatin conformation, Text mining, Disease network

## Abstract

**Background:**

Smith-Magenis syndrome (SMS) is a developmental disability/multiple congenital anomaly disorder resulting from haploinsufficiency of *RAI1.* It is characterized by distinctive facial features, brachydactyly, sleep disturbances, and stereotypic behaviors.

**Methods:**

We investigated a cohort of 15 individuals with a clinical suspicion of SMS who showed neither deletion in the SMS critical region nor damaging variants in *RAI1* using whole exome sequencing. A combination of network analysis (co-expression and biomedical text mining), transcriptomics, and circularized chromatin conformation capture (4C-seq) was applied to verify whether modified genes are part of the same disease network as known SMS-causing genes.

**Results:**

Potentially deleterious variants were identified in nine of these individuals using whole-exome sequencing. Eight of these changes affect *KMT2D*, *ZEB2*, *MAP2K2*, *GLDC*, *CASK*, *MECP2*, *KDM5C*, and *POGZ*, known to be associated with Kabuki syndrome 1, Mowat-Wilson syndrome, cardiofaciocutaneous syndrome, glycine encephalopathy, mental retardation and microcephaly with pontine and cerebellar hypoplasia, X-linked mental retardation 13, X-linked mental retardation Claes-Jensen type, and White-Sutton syndrome, respectively. The ninth individual carries a de novo variant in *JAKMIP1*, a regulator of neuronal translation that was recently found deleted in a patient with autism spectrum disorder. Analyses of co-expression and biomedical text mining suggest that these pathologies and SMS are part of the same disease network. Further support for this hypothesis was obtained from transcriptome profiling that showed that the expression levels of both *Zeb2* and *Map2k2* are perturbed in *Rai1* ^–/–^ mice. As an orthogonal approach to potentially contributory disease gene variants, we used chromatin conformation capture to reveal chromatin contacts between *RAI1* and the loci flanking *ZEB2* and *GLDC*, as well as between *RAI1* and human orthologs of the genes that show perturbed expression in our *Rai1* ^–/–^ mouse model.

**Conclusions:**

These holistic studies of *RAI1* and its interactions allow insights into SMS and other disorders associated with intellectual disability and behavioral abnormalities. Our findings support a pan-genomic approach to the molecular diagnosis of a distinctive disorder.

**Electronic supplementary material:**

The online version of this article (doi:10.1186/s13073-016-0359-z) contains supplementary material, which is available to authorized users.

## Background

Smith-Magenis syndrome (SMS; MIM #182290) is a rare genomic disorder with a prevalence of 1 in 15,000. It is associated with specific craniofacial dysmorphology, developmental delay (DD), moderate to profound intellectual disability (ID), and self-injurious and stereotypic behaviors [1, 2]. SMS individuals show sleep disturbance with frequent daytime napping and night-time awakenings. They display restricted interest, obsessive thinking, and social responsiveness scale scores consistent with autism spectrum disorder (ASD) [3]. They repetitively mouth objects, rock, spin, or twirl their body, and grind their teeth [4]. This distinctive profile is complemented by specific lick and flip and self-hug behaviors, as well as attachment to people [5–7]. Challenging behaviors such as self-injuries, physical aggression, and destructive behavior are significantly more prevalent in SMS than in ID with mixed etiologies [8]. Self-injuries are present in 70–97 % of individuals and include polyembolokoilamania (insertion of foreign objects into bodily orifices) and onychotillomania (pulling out finger and toe nails). Unusual behaviors can comprise poking others’ eyes, forceful hugging, and punching fists through walls and windows.

Whereas SMS is classically associated with a deletion within cytogenetic G-band 17p11.2 that includes the *RAI1* gene (about 90 % of individuals) or a nucleotide variant in that gene (about 5 %) [1, 9–12], some reports suggested genetic heterogeneity as SMS-like individuals were found to recurrently harbor deletions of the 2q37.3 or 2q23.1 cytobands encompassing *HDAC4* and *MBD5*, respectively [13–15]. Similarly, *PITX3* was proposed to be responsible for the SMS-like neurobehavioral abnormalities observed in an individual [16].

Here we use recent advances in genome sequencing technologies to further assess the genetic heterogeneity of SMS and the possible clinical overlap of this syndrome with other intellectual disability and cognitive dysfunction disorders, as some of the seemingly characteristic phenotypic features are non-discriminating among ID syndromes. We also evaluate the pertinence of network interactions and provide experimental data in support of potential molecular diagnoses.

## Methods

### Enrollment

Each of the 149 patients was clinically assessed by their respective physicians. Patients were diagnosed as potentially affected by SMS through clinical assessment. Briefly, all individuals presented intellectual disability and/or developmental delay, and the majority (>75 %) also had sleep disturbances, stereotypies, or other endophenotypes common to SMS (e.g. distinctive facial features, tantrums, self-injurious behaviors, onychotillomania). The clinical presentation of SMS is heterogeneous; therefore, the indication of SMS by a clinician can be either premature in the case of a young infant or possibly a misdiagnosis in an individual with behavioral issues and ID.

### Detailed SMS patients’ phenotypes

The detailed phenotype descriptions of 13 of the 15 patients without *RAI1* genetic alteration are described in Additional file 1: Supplementary text and Additional file 2: Table S1. The remaining two individuals had no clinical data available.

### Array comparative genomic hybridization

Targeted chromosome 17p array comparative genomic hybridization (aCGH) analyses were carried out on each proband as previously reported [17]. Additional genome-wide aCGH was conducted on each person using Baylor Miraca Genetics Laboratory design version 10.1, an Agilent 180 K oligo array. All array data were analyzed as previously described [18].

### Exome sequencing

To uncover genetic variants associated with the abnormalities shown by the 15 patients without *RAI1* genetic alteration, we performed whole-exome sequencing of DNA extracted from blood of the proband and both their parents whenever possible (eight trios) at the Baylor College of Medicine (BCM) Human Genome Sequencing Center (HGSC) via the Baylor-Hopkins Center for Mendelian Genetics. Exomes were captured and sequenced on an Illumina HiSeq platform using previously described methods [19]. Sequence analysis was performed using the HGSC Mercury analysis pipeline (https://www.hgsc.bcm.edu/software/mercury) [20]. Variants were filtered based on inheritance patterns including autosomal recessive, X-linked, and de novo/autosomal dominant. Variants with MAF < 0.05 in control cohorts (Atherosclerosis Risk in Communities (ARIC, https://www2.cscc.unc.edu/aric/), 1000 Genomes project (http://www.1000genomes.org/), the NHLBI Exome Sequencing Project (http://evs.gs.washington.edu/EVS/), and our internal BCM control database of > 5000 exomes generated as a member of the Centers for Mendelian Genomics) [21] and predicted to be deleterious by SIFT10 and/or PolyPhen were prioritized [19]. Sanger sequencing confirmed putatively causative variants and their familial segregation.

The sequencing variants identified in this manuscript were deposited in ClinVar (https://www.ncbi.nlm.nih.gov/clinvar/).

### Modeling

The primary sequence of each candidate protein was loaded in Swiss-PdbViewer aligned onto suitable modeling templates retrieved from SWISS-MODEL and superposed in three-dimensional (3D) space using Swiss-PdbViewer [22, 23]. Each variant was modeled in the context of the overall 3D structure to evaluate its potential impact with respect to protein folding, as well as to position of known disease-associated variants. We also assessed if missense variants perturbing the protein function clustered in 3D around key regions of the protein [24].

The ZEB2 Zinc finger residues 995–1078 were modeled using the pdb entry 1mey as template [25]. MAP2K2 was modeled using both MAP2K2 (pdb entry 1s9i 3.2A resolution [26] and MAP2K1 (pdb entry 3eqi, 1.9A resolution) structures [27]. The GLDC residues were aligned on the *Synechocystis sp*. glycine decarboxylase model PCC 6803 (pdb entry 4LHD) [28]. To model the CASK variants, two partial CASK crystal structures (pdb entries 1kwa, chain A [29] and 1kgd, chain A (http://www.ncbi.nlm.nih.gov/pubmed/11729206?dopt=Abstract)) covering residues 487–572 and 739–914, respectively, were superposed on the crystal structure of PALS1/Crb (pdb entry 4wsi [30]) that present 35 % identity with CASK.

### Literature mining

Because literature resources do not use entity name in a consistent way, we first checked each gene identifier by using UniProtKB (http://www.uniprot.org) or HUGO Gene Nomenclature Committee (HGNC) database (http://www.genenames.org) in order to retrieve the recommended/approved name, short name(s), alternative and synonymous name(s) if any for each targeted gene, as well as the name(s) of the encoded protein. These were used as singleton and/or pairwise strings to extract information from various literature resources: PubMed (http://www.ncbi.nlm.nih.gov/pubmed), Google Scholar, iHOP (http://www.ihop-net.org/UniPub/iHOP/), and EVEX (http://evexdb.org/), to cite here the original source of reference for this project. The obtained results were curated and the reported relationships were visualized using Cytoscape (3.2.1; http://www.cytoscape.org/). The connectivity was assessed using the Knet-function, which is based on the adaptation of spatial statistics concepts to network analysis proposed in [31]. The statistical significance of the obtained Knet-function value was calculated with respect to a population of permuted networks (*n* = 10^6^) derived from the original prior knowledge network. It is worth noting here that the connectivity is not only based on direct but also on indirect connections through shortest paths.

### Identification of RAI1 interacting proteins

We identified ZBTB17/MIZ1 and BRD2 as likely interactors for RAI1 with a yeast two-hybrid assay. The yeast two-hybrid assays were performed in collaboration with the company Proteinlinks. Briefly, two fragments of the carboxyl-terminus of mouse *Rai1* (a.a 1246–1841 and a.a. 1246–1890) were cloned into pCWX200 as baits. Around 10 million independent complementary DNA (cDNA) library clones (10× library coverage) were screened for protein–protein interactions with both baits. We cultured the Y304 yeast strain on galactose selective medium without leucine, histidine, trytophan, and uracil. Positive clones were replicated onto the four selective plates and examined with URA3 (or LEU2) and LacZ reporters. From this analysis, we identified ZBTB17/MIZ1, BRD2, and SOGA3 as reasonable candidates (at least two clones, supported by both baits) for RAI1 interaction candidates. These interactions were further assessed using co-immunoprecipitation (co-IP) analysis in HEK293 cells. Full-length *Rai1* was cloned in pCMV-3xFLAG vector while the three candidates were cloned into pCMV-HA vectors to confirm the yeast two-hybrid results. Lysate from the co-transfected HEK293 cells (RAI1 and one of the candidates) was purified with EZview FLAG-M2 beads (Sigma) and analyzed with rat anti-HA (Abcam) on western blot. The interaction between RAI1 and ZBTB17/MIZ1 was confirmed by co-IP, however *BRD2* did not express well enough on western blot, and *SOGA3* was too sticky to conduct co-IP with, as it bound to the beads in the absence of FLAG-RAI1 (Additional file 3: Figure S1).

### Embryo collection and RNA extraction

Mice were housed in standard specific pathogen-free conditions. All animal studies were conducted under protocols approved by the Baylor Institutional Animal Care and Use Committee and followed NIH guidelines. Timed matings between *Rai1* heterozygous females and males in F2 generation in the C57BL/6 *Tyr* ^*c-Brd*^ and 129SvEv mixed genetic background were implemented to generate *Rai1* ^*–/–*^ embryos. To harvest embryos, pregnant females were sacrificed by cervical dislocation and the embryos were dissected from the uterus in ice-cold phosphate buffered saline (PBS) solution. Similar sized embryos at 10.5 days post conception (dpc) were collected in 1.5 mL Eppendorf tubes, frozen immediately in liquid nitrogen, and stored in –80 °C. Portions of the yolk sac were saved for genotyping as described previously [32]. For RNA extraction, the whole embryos were homogenized in Trizol and RNA was extracted according to the manufacturer’s instructions (Invitrogen) followed by purification on columns using an RNeasy mini kit (Qiagen Sciences, Germantown, MD, USA). The RNA integrity, concentration, and overall quality were tested with an Agilent Bioanalyzer 2100 and a NanoDrop ND-1000 spectrophotometer.

### Microarray processing and analysis

A total of 5–10 μg of total RNA from each individual embryo of three *Rai1* ^*–/–*^ at 10.5 dpc and three wild-type controls were used to produce complementary RNA (cRNA) target microarray transcriptome analyses. Embryos at 10.5 dpc were chosen because *Rai1* functions during this stage as indicated by its strong expression and embryonic lethality of *Rai1* ^*–/–*^ embryos from 7.5 to 18.5 dpc [32]. In addition, the size of the *Rai1* ^*–/–*^ embryos at 10.5 dpc is comparable to that of their wild-type littermates whereas the few surviving *Rai1* ^*–/–*^ mice at birth are significantly smaller than the wild-type [32]. The integrity and quality of the extracted RNAs were assessed on a 2100 Bioanalyzer (Agilent, Santa Clara, CA, USA). The target was generated using a reverse transcription reaction to produce cDNA (SuperScript Choice System, Gibco), which was subsequently subjected to in vitro transcription with biotinylated cytidine-5′-triphosphate and uridine-5′-triphosphate using ENZo BioArray High Yield RNA Transcript Labeling kit to produce biotinylated cRNA. The target was then fragmented and hybridized to Affymetrix Mouse Genome 430 2.0 Array GeneChips (Affymetrix, Santa Clara, CA, USA) in duplicates using an Affymetrix GeneChip Fluidics Station 400. The arrays were stained with phycoerythrin-coupled avidin and scanned using a GeneArray Scanner 3000. The resultant output was analyzed using Affymetrix Microarray Suite software and examined for excessive background or evidence for RNA degradation. The chips were assessed by scaling factor, average background, percent of probe sets that are present, number of probes present, and the 3′-end to 5′-end probe intensity ratio for housekeeping probe sets (*β-actin* and *GAPDH*), as well as the number of probes present for the “spiked in” probe sets (*BioB*, *BioC*, *BioD*, and *Crex*). All the chips were of good quality, which is further supported by the observations that they have similar RNA degradation patterns and the chips were well replicated within the same genotype group as shown by scatter plot analyses. The criteria for genes differentially expressed are that the log ratio of the normalized expression values in the *Rai1* deficient embryos versus the controls is > 0.5 and the *P* value < 0.05, which empirically gives a very low false detection rate (FDR). The probe sets with very low expression values were filtered out. We analyzed the chromosomal position of all the regulated genes using the chromosomal coordinates within recent genome assemblies of the mouse. The array data were analyzed using the GC-RMA program to estimate the expression measures from the probe level data [33]. The program corrects the background, normalizes the raw perfect match data using the quantile normalization method, and summarizes the probe values to probe set values (expression values, one per probe set per chip), in log_2_ scale. The fold change for each probe is the log ratio of average expression value in the mutant samples divided by that in the wild type controls. The fold change is considered to be significant if *P* ≤ 0.05.

### Reverse transcription polymerase chain reaction (RT-PCR) validation

For RT-PCR validation of relevant expression targets, 1 μg of total RNA (intact by gel and measured by NanoDrop) was used for RT reactions using the Quanta qScript cDNA synthesis kit. Three separate RT reactions were performed using RNA from both a *Rai1* ^*–/–*^ embryo and a wild-type control littermate. The RT reactions and a non-RT reaction using wild-type RNA as well as a water-only control were then run on a gel and all reactions containing both RNA and RT had similar patterns and intensity. A total of 1 μL of each RT reaction was used for subsequent PCR reactions. Primers for PCR were designed to transcript regions of *Zeb2*, *Map2k2*, and *Rai1* using the UCSC browser version of GRCm38/mm10. The primers (from 5′ to 3′) are as follows:

*Zeb2-*8R: ATGTGAACTGTAGGACCCAGAATGA

*Zeb2-*7 F: CTTCAAGTACAAGCACCACCTGAA

*Map2k2*-2 F: TGAGAGGATCTCAGAGCTGGGT

*Map2k2*-3R: ACTCGTGCAGCACCTGCA

*Rai1-*4 F: ATGTATCCACACCTACCACTACCCAT

*Rai1*-5R: ACTTCAAAGTAAAATTCTCCTCAATGAACGT

### 4C-seq and 3C-PCR validation assays

Circularized chromosome conformation capture (4C) libraries were prepared from lymphoblastoid cell lines (LCLs) of two age-matched female control individuals. Briefly, LCLs were grown at 37 °C. 5 × 10^7^ exponentially growing cells were harvested and crosslinked with 1 % formaldehyde, lysed, and cut with DpnII, a 4-cutter restriction enzyme that allows higher resolution [34, 35]. After ligation and reversal of the crosslinks, the DNA was purified to obtain the 3C library. This 3C library was further digested with NlaIII and circularized to obtain a 4C library. The inverse PCR primers to amplify 4C-seq (4C combined with multiplexed high-throughput sequencing) templates were designed to contain Illumina adaptor tails, sample barcodes, and viewpoint-specific sequences. The selected viewpoint maps within the 5′ portion of the first intron of the *RAI1* gene (700 bp from the donor site of exon 1), a region enriched in DNaseI hypersensitive and transcription factor binding sites [36]. It corresponds to the closest suitable DpnII fragment relative to the transcriptional start sites of the targeted gene. The sequence of the 4C-seq primers is reported in Additional file 2: Table S2. We amplified at least 1.6 μg of 4C template (using about 100 ng of 4C template per inverse PCR reaction, for a total number of 16 PCRs). We multiplexed the two 4C-seq templates in equimolar ratios and analyzed them on a 100-bp single-end Illumina HiSeq flow cell. The numbers of raw, excluded, and mapped reads for each LCL sample are detailed in Additional file 2: Table S3.

To validate selected physical interactions and loop formations between non-neighboring chromatin fragments, 5 × 10^7^ exponentially growing cells were used in conjunction with our 3C protocol as described [37]. We tested primers positioned on the chromosome *17p11.2* sense strand 5′ to 3 for the cis-interactions and primers designed at 9p24.1 compared to control 16p11.2 region for the trans-interactions (Additional file 3: Figure S2 with primers tables). The presence of physical interactions was determined by PCR amplimer production. Control PCRs included no input (“water”) as well as DNA from chromatin digested with DpnII but without the subsequent religation step (“- Ligase”) (Additional file 3: Figure S2).

### 4C-seq data analysis

4C-seq data were analyzed as previously described [34, 35, 37] through the 4C-seq pipeline available at http://htsstation.epfl.ch/) [38] and visualized with gFeatBrowser (http://www.gfeatbrowser.com). Briefly, the multiplexed samples were separated, undigested, and self-ligated reads removed. Remaining reads were aligned and translated to a virtual library of DpnII fragments. Read counts were then normalized to the total number of reads and replicates combined by averaging the resulting signal densities (Additional file 3: Figures S3 and S4). The local correlation between the profiles of the two samples per viewpoint was calculated (Spearman correlation: 0.83). The combined profiles were then smoothed with a window size of 29 fragments. The region directly surrounding the viewpoint is usually highly enriched and can show considerable experimental variation, thereby influencing overall fragment count. To minimize these effects, the viewpoint itself and the directly neighboring “undigested” fragment were excluded during the procedure. In addition to this filtering, we modeled the data to apply a profile correction similar to the one described in [39] using a fit with a slope -1 in a log-log scale [40]. Significantly interacting regions were detected by applying a domainogram analysis as described [41]. We selected BRICKS (Blocks of Regulators In Chromosomal Kontext) with a *p* value threshold < 0.01 for both “cis” and “trans” interactions, and annotated the BRICKs overlapping genes as well as the closest upstream and the closest downstream genes, in a window of +/– 500 kb. The 4C libraries used to perform the circular PCR with RAI1 viewpoint’s primers had been previously tested in [35], with seven additional viewpoints’ primer pairs. The BRICKs genes GTDC1 and KDM4C (and the flanking genes ZEB2 and GLDC) were not called as significantly interacting regions for any of these viewpoints (see [35]; Additional file 2: Tables S6–S12). The raw sequencing files are available at GEO under accession number GSE83420.

### Enrichment analyses

Gene annotation was obtained through BioScript (http://gdv.epfl.ch/bs). Protein interaction networks for BRICKs genes were determined using STRING (Search Tool for the Retrieval of Interacting Genes/Proteins) v9.1 (http://string-db.org/) [42]. We exploited GO with Enrichr (http://amp.pharm.mssm.edu/Enrichr/) to assess if the chromatin-contacted genes were enriched in specific pathways and genes associated with Mendelian diseases and GIANT (http://giant.princeton.edu/) and Genemania (http://www.genemania.org/) to test tissue-specific functional interactions and produce association networks, respectively [43–46]. The significance of the connectivity of the GIANT co-expression networks was assessed as described for the literature-mining network (see above). We used Enrichr Chromosome Location tool and BRICKS count in different window sizes (5 Mb, 1 Mb, and 500 kb) to determine whether any cytogenetic band other than 17p11.2 was enriched for BRICKS. Other than 17p11.2, we identified significant enrichments at cytobands 17p12, 17p13, and 2q22, where the gene *ZEB2* is located.

### Hi-C data

Hi-C matrices from Rao et al. [47] were prepared by first applying a KR normalization to the 5 kb and 100 kb resolution observed matrices and then by dividing each normalized score by the expected one extracted from the KR expected file (as described previously in section II.c of the Extended Experimental Procedures of reference [47]). KR expected values less than 1 were set to 1 to avoid long-distance interaction biases. HiC matrices from Dixon et al. [48] were generated from the normalized datasets at a 40 kb resolution and transformed to a 400 kb resolution by summing the contacts observed in 10 × 10 sub-matrices. Expected vectors represent the mean number of contacts observed at a given distance and were used to calculate the observed/expected matrices.

## Results

### Clinical and molecular findings

Through physicians from a large network of medical genetics centers, we enrolled a cohort of 149 individuals presenting with a constellation of SMS features. High-density 17p11.2 aCGH and Sanger sequencing of *RAI1* showed that 134 out of 149 individuals presented a genetic or genomic alteration of the *RAI1* gene [9, 11, 17, 49–52], 96/134 (72 %) individuals carried the classic recurrent 3.7 Mb SMS deletion, ten (7.5 %) contained an uncommon recurrent 1 (UR1) or UR2 rearrangement, 24 (18 %) a non-recurrent *RAI1* deletion, and four (3 %) had a de novo variant in *RAI1* [9, 11, 49, 52, 53] (Additional file 2 Table S1). Whereas these proportions are similar to published results [12, 54], it is likely that some clinicians did not refer individuals with SMS features who were negative for SMS molecular diagnosis (via aCGH or fluorescence in situ hybridization, FISH) or who were positive for another potentially causative CNV, for example 1p36 deletion syndrome [15, 55, 56] that shares multiple similarities with SMS. Indeed, many individuals were molecularly diagnosed prior to sample submission. Consistent with this hypothesis, a separate study identified mutations affecting *RAI1* in only 30 % of participants with a suspected diagnosis of SMS [12].

The remaining 15 individuals (10 %) showed no discernable perturbation of the *RAI1* gene. The 13 with available clinical data presented the following classical SMS features: ID (12/12), DD (13/13), sleep disturbances (8/10), and/or self-injurious behavior (10/11), in particular onychotillomania (6/7) (Additional file 1: Supplementary Text, Additional file 1: Table S1). To identify the underlying cause of the phenotypes of these 15 individuals, the probands and their parents when available (eight cases) were subjected to high-resolution genome-wide aCGH and whole-exome sequencing. We identified potentially causative variants in ten individuals (Additional file 2: Table S1). These were grouped into five categories: (1) a 47, XYY karyotype (subject BAB2492); (2) de novo variants in *ZEB2* (BAB2386), *CASK* (BAB2540), *KMT2D* (BAB2319), and *JAKMIP1* (BAB2451); (3) compound heterozygote variants in *GLDC* (BAB4947); (4) a *MECP2* variant in a woman with random X-inactivation (BAB2552) inherited from the individual’s mother, who presented with a skewed X-inactivation pattern (away from this allele) in her blood (Additional file 3: Figure S5); and (5) variants in *POGZ* (BAB2330, variant not maternally inherited), *MAP2K2* (BAB2474), and the X-linked *KDM5C* (BAB2293), the origins of which could not be assessed. We confirmed the segregation of sequence variants in available family members by Sanger sequencing.

Individual BAB2330 and four other carriers of heterozygous truncating variants in *POGZ* allowed the recent description of a new syndromic form of intellectual disability [57, 58]. We compared the phenotype of the remaining individuals (Additional file 1: Supplementary Text, Table 1, Additional file 2: Table S1) with those associated with the identified molecular diagnoses, including 47,XYY, Mowat-Wilson syndrome (MOWS; OMIM#235730), mental retardation and microcephaly with pontine and cerebellar hypoplasia (MICPCH; OMIM#300749), Kabuki syndrome-1 (KABUK1; OMIM#147920), glycine encephalopathy (GCE; OMIM#605899), X-linked syndromic mental retardation 13 (MRXS13; OMIM#300055), cardiofaciocutaneous syndrome (CFC4; OMIM #615280), and X-linked syndromic mental retardation Claes-Jensen type (MRXSC; OMIM#300534) (Additional file 2: Table S4). While we observed distinct clinical features in some individuals (e.g. macrocephaly and seizures in the carriers of variants in *KDM5C* and *GLDC*, respectively (Additional file 1: Supplementary text)), features specific to SMS [59] are present in a sufficient number of probands (Additional file 2: Table S1). This allows us to hypothesize that in some cases the molecular diagnosis hinted at potential underlying genetic heterogeneity for SMS rather than misdiagnoses of other syndromes, and that some of the 47,XYY, MOWS, MICPCH, KABUK1, GCE, MRXS13, CFC4, and MRXSC syndromes have a greater clinical phenotypic variability than anticipated. This prompted investigation of the presumptive effect of the variants on the encoded proteins and molecular perturbations that may underlie the observed phenotypic manifestations (summarized in Table 1).

**Table 1:**
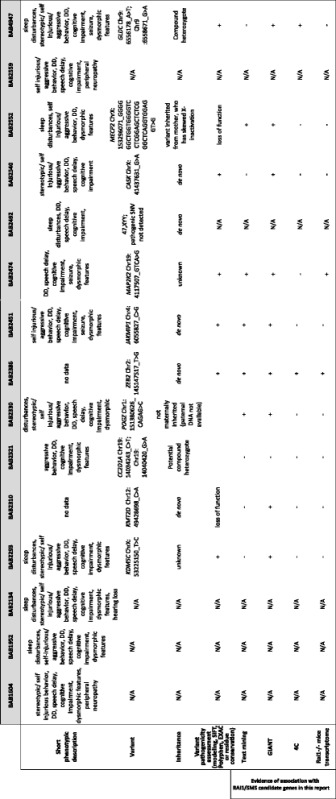
Summary of clinical phenotypes of SMS patients without RAI1 alteration, variants assessment, and evidence of association with RAI1presented in this paper

### Variant analysis and modeling

The variants identified in *KMT2D* (p.E3418X) and *MECP2* (p.P389fsX) are predicted to be loss-of-function alleles, which are likely pathogenic alleles as *KMT2D* and *MECP2* are “extremely intolerant” and “intolerant” to loss-of-function variation according to the Exome Aggregation Consortium database version 0.3 (http://exac.broadinstitute.org) [10.2015] (pLI = 1.0 and 0.7, respectively) and as analogous loss-of-function variants in *KMT2D* and *MECP2* were identified in KABUK1 [60] and MRXS13 [61] individuals, respectively (Additional file 2: Table S5 and S6). Additionally, the de novo variant in the candidate gene *JAKMIP1* (p.D586H) occurs in a highly conserved residue and is predicted to be deleterious to the protein structure. *JAKMIP1* is “extremely intolerant” to loss-of-function variation according to ExAC (pLI = 0.99). When possible, we used X-ray structures and/or cryo-EM modeling to obtain a 3D representation of the remaining encoded proteins and compared the variants we identified with those previously reported in MOWS, MICPCH, GCE, CFC4, and MRXSC individuals (Additional file 2: Table S7–S11). By and large, these models suggest that the variants identified in the current study are detrimental to the encoded proteins: (1) the ZEB2 p.H1049P variant substitutes a residue that participates in the coordination of the Zn^++^ atom of one of the Zinc fingers, similar to the variant p.H1045R identified in a MOWS individual (Additional file 3: Figure S6A; Additional file 2: Table S11); (2) the MAP2K2 p.D69del variant removes one of the two aspartic acid residues involved in the binding of a Ca^++^ ion in the conserved GELKDD loop (Additional file 3: Figure S6B); (3) the GLDC p.L726Q and p.P647L variants likely affect the packing of the encoded protein in the neighborhood of the catalytic lysine K754 residue similar to the 61 missense variants identified in GCE individuals (Additional file 3: Figure S6C, Additional file 2: Table S8); and (4) the CASK p.R489W variant places a bulky tryptophan sidechain that cannot be accommodated in the structure without changing the molecular surface (Additional file 3: Figure S6D). The possible impact of the KDM5C p.K1023R variant on this conserved position (Additional file 3: Figure S7) could not be evaluated as no template is available for this region.

### The identified rare variants affect Rai1-associated genes

**Fig. 1:**
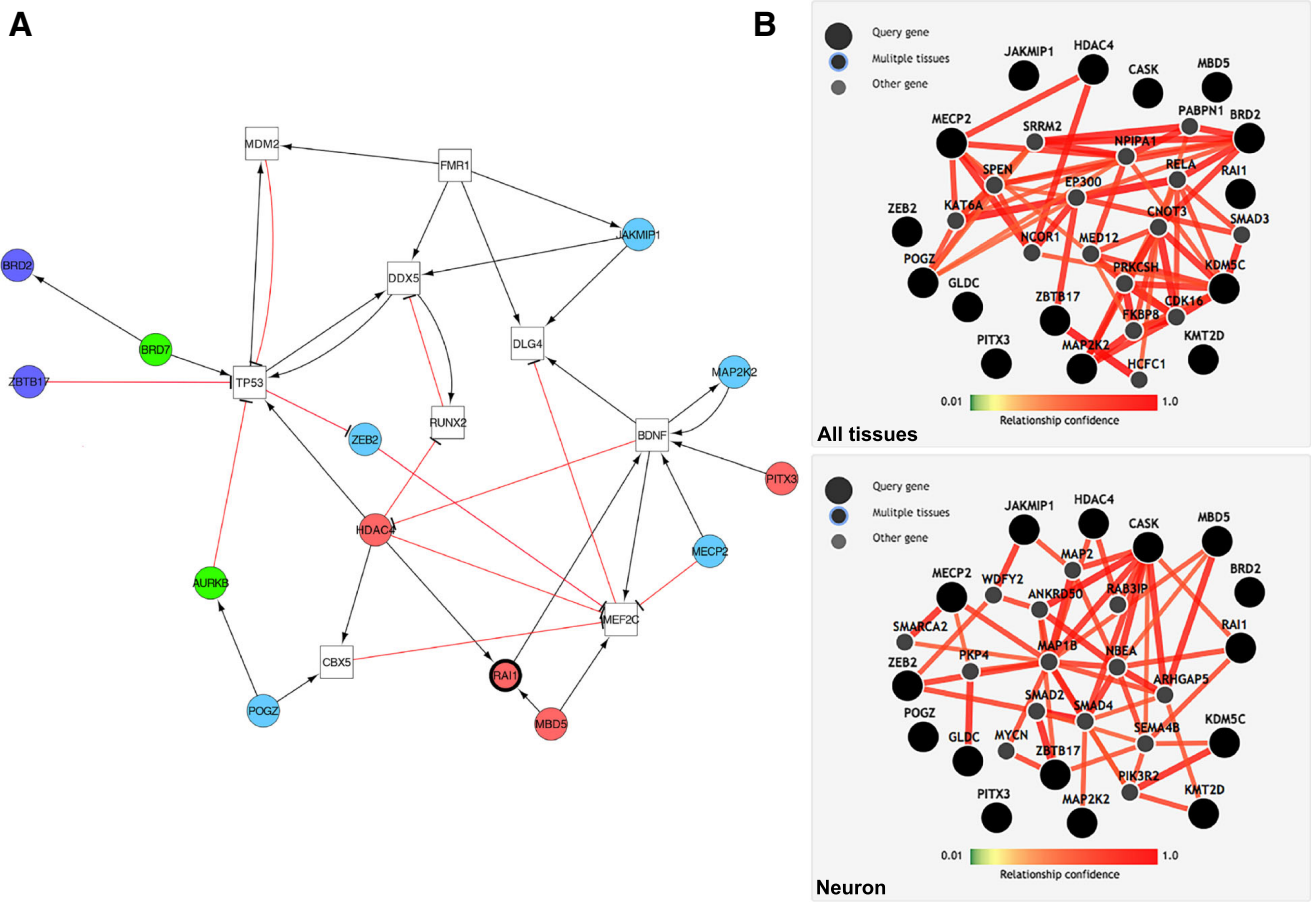
*RAI1* Molecular interactions. **a** Literature-defined molecular interactions. Genes interactions network obtained combining literature text-mining resources (i.e. PubMed, Google Scholar, iHOP, and EVEX) and visualized using Cytoscape. Nodes are *colored* depending on the role of the gene: genes associated with SMS phenotypes in *red* (*RAI1* is highlighted with a thicker outline); *RAI1* interactors identified in yeast two-hybrid screens in *dark blue*; genes with rare possibly damaging variants in SMS patients identified in this report in *light blue*; “extra nodes” required to make connection are shown as *white squares* (maximum two “extra nodes” allowed), while “extra nodes” found in the 4C BRICKs of the *RAI1* viewpoint are depicted in *green*. **b** Co-expression-based molecular interactions. Tissue-specific functional interaction network built using GIANT (Genome-scale Integrated Analysis of gene Networks in Tissues, http://giant.princeton.edu/) in “all tissues” (*top panel*) versus “neuron” (*bottom panel*), with minimum relationship confidence = 0.5 and maximum number of genes = 15. *RAI1*, *HDAC4*, *PITX3*, *MBD5*, *BRD2*, *MIZ1/ZBTB17*, *CASK*, *KMT2D*, *MECP2*, *GLDC*, *KDM5C*, *MAP2K2*, *POGZ*, and *ZEB2* were used as queries

We next proceeded to test the hypothesis that genes mutated in individuals with SMS-like features were associated with *RAI1.* To challenge this assumption, we first assessed if *HDAC4*, *MBD5*, and *PITX3*, three genes previously reported to be associated with SMS phenotypes [13, 14, 16], *BRD2* and *ZBTB17* (a.k.a. *MIZ1*), two genes encoding high-confidence RAI1 interactors we identified by two-hybrid assay (see “Methods”) and *JAKMIP1*, *ZEB2*, *CASK*, *KMT2D*, *GLDC*, *MECP2*, *MAP2K2*, *POGZ*, and *KDM5C*, the nine genes identified here, were part of a *RAI1* functional network. Manual curation of the literature revealed single or double edges functional relationships between 13 of these 15 genes, allowing a maximum of two “extra” connecting nodes. This network includes *JAKMIP1*, *ZEB2*, *MECP2*, and *MAP2K2* (Fig. 1a; Table 1), indicating that may have uncovered a “disease network” as previously described [62]. The significance of the observed connectivity (*P* = 0.0167 was assessed adapting spatial statistics concepts to network analysis [31] (see “Methods”). Second, we used the GIANT database (Genome-scale Integrated Analysis of gene Networks in Tissues [63]) to assess whether these 14 genes form a functional network and eventually capture tissue-specific functional interactions. When considering GIANT data from neurons, *CASK* functions as a provincial hub with nine edges and 12 genes *(JAKMIP1*, *CASK*, *GLDC*, *HDAC4*, *KDM5C*, *KMT2D*, *MAP2K2*, *MBD5*, *MECP2*, *RAI1*, *ZBTB17*, and *ZEB2*) of the 15 assessed are connected (*p* = 0.0439), further supporting the notion of “disease network” as, in particular, *HDAC4* and *MBD5*, two of the three genes previously associated with SMS-like phenotypes, are included [13–15]. Eight out of 14 genes (*BRD2*, *HDAC4*, *KDM5C*, *MAP2K2*, *MECP2*, *POGZ*, *RAI1*, and *ZBTB17*) including, in particular, the two genes, *BRD2* and *ZBTB17*, encoding high-confidence RAI1 interactors, are functionally linked in the “all tissue” network (*p* = 0.00814; Fig. 1b; Table 1;). Furthermore, when *RAI1* is used as single query gene, *RAI1* and *CASK* are directly linked in the resulting gene network but, again, specifically in neurons (Additional file 3: Figure S8). These results and the data extracted from the literature suggest that at least some of the eight genes with variants potentially causing SMS-like phenotypes could possibly be causative as they are functionally associated with *RAI1*.

To gain further insight about the genes regulated by *Rai1* during mouse embryonic development, we performed microarray analysis on total RNA prepared from three 10.5 dpc *Rai1* ^*–/–*^ embryos and from three of their wild-type littermates. The two *Rai1* transcripts present on the array are significantly downregulated in *Rai1* ^*–/–*^ embryos compared to wild-type littermates (e.g. the AK013909 transcript with a fold change of 6.2 shows the largest downregulation among the 45,037 assessed probe sets). In fact, the expression values for both transcripts are within background levels in the *Rai1* ^*–/–*^ embryos, indicating that both transcripts are not expressed in the *Rai1* ^*–/–*^ mutants and further corroborating the contention that the engineered *Rai1* mutant allele is a complete null allele [32]. In total, 142 and 157 probe sets showed an over twofold increase or decrease, respectively (Additional file 2: Table S12; see “Methods”) in the mutant mice when compared to wild-type littermates. Consistent with the hypothesis that genes potentially causative of the SMS-like phenotypes are functionally associated to or transcriptionally regulated by *RAI1*, the expression levels of both *Zeb2 (*ENSMUSG00000026872) and *Map2k2 (*ENSMUSG00000035027*)* were perturbed in *Rai1* ^–/–^ mice (Additional file 2 Table S12). These expression arrays results were subsequently confirmed by RT-PCR (Additional file 3: Figure S9). We then assessed the chromosomal position of the dysregulated genes. The enrichment score using a Pearson Chi-square goodness of fit statistic indicated that they showed a biased chromosome distribution with 22 % of the genes downregulated and 26 % of the genes upregulated in the *Rai1* mutants mapping to mouse chromosome 11 (MMU11) where the *Rai1* gene resides. Less than 5 % of the differentially expressed genes are located on any chromosome other than MMU11. This enrichment on MMU11 for downregulated and upregulated genes in *Rai1* ^*–/–*^ embryos is reminiscent of our previous finding that the engineered MMU11 deletion and reciprocal duplication that mimic SMS and Potocki-Lupski syndrome rearrangements were associated with a MMU11-wide transcriptome perturbation in the five assessed adult male tissues [64].

**Fig. 2:**
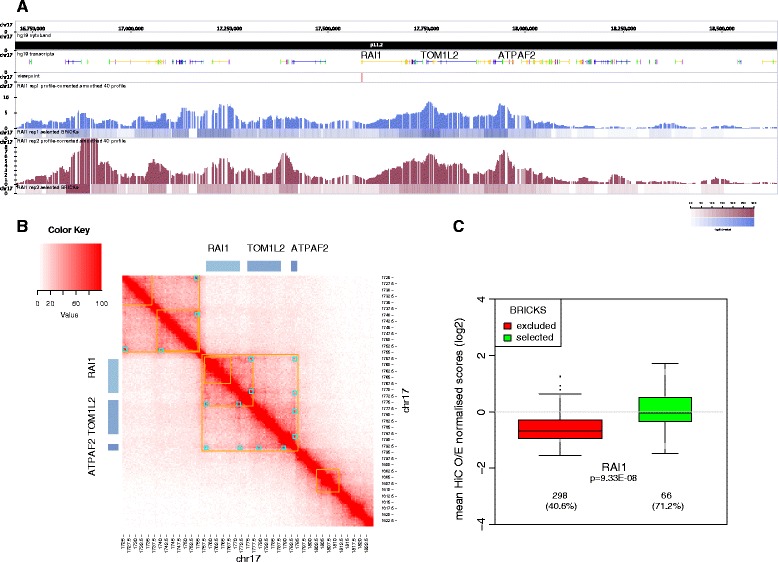
4C interactions profile of *RAI1* and comparison with Hi-C interactions profiles locally and globally. **a** (*Panels from top to bottom*). Transcripts: The structure of the transcripts mapping within human 17p11.2 cytoband from approximately 16.5 Mb to 18.5 Mb are indicated, in particular those of the *RAI1*, *TOM1L2*, and *ATPAF2* genes. Viewpoint: The *red tick* shows the mapping position of the *RAI1* viewpoint used in the 4C experiments. PC/BRICKs: Smoothed and profile-corrected 4C signal (*upper part of each panel*) and BRICKs (*lower part*) identified for each replicate (*blue* and *burgundy*). The corresponding BRICKs significance *heatmap color legend* is shown in the *bottom right corner*. **b** High resolution Hi-C chromosome conformation capture results obtained with the GM12878 LCL within the chromosome 17 17.25-18.22 Mb window (5 kb resolution). *Yellow* and *light blue squares* highlight the contact domains and peaks identified in [47], respectively. The position of the *RAI1*, *TOM1L2*, and *ATPAF2* genes is indicated. **c** Distribution of Hi-C scores in selected (FDR1%) versus non-selected BRICKS (FDR10%). Virtual 4C-seq tracks were generated for the *RAI1* viewpoint from the GM12878 Hi-C results published in [47] (5 kb resolution) by extracting the Hi-C vectors from the KR normalized observed/expected matrices. BRICKS found with the viewpoint were quantified by the mean Hi-C signals. The *p* value of the two-sided t-test is reported for the comparison, together with the number of Hi-C bins and the % of non-NA bins

Chromatin architecture can similarly be exploited to identify genes that belong to the same pathway. Long-range chromatin contacts, which bring genes in close proximity to regulatory sequences, have been shown to be necessary for co-transcription of biologically related and developmentally co-regulated genes [65, 66]. We recently showed the pertinence of this approach by documenting that genes associated with ASD and head circumference phenotypes were linked by chromatin loops. As a third approach to assess if *RAI1* is biologically related to the eight genes identified in the SMS-like individuals, we used an adapted version of the 4C method [35, 37, 67–69] to identify chromosomal regions that physically associate with the *RAI1* “viewpoint.” We independently analyzed the local pattern of chromosomal interactions in LCLs of two control individuals (Additional file 3: Figures S10, S3, and S4, see “Methods”). Genome-wide, we detected 153 significant BRICKs (FDR ≤ 1 %), i.e. 3D interacting genomic fragments (see “Methods,” Additional file 2: Table S13), encompassing 147 genes. Within the 66 (43 %) intrachromosomal BRICKs, we identified, in particular, two genomic intervals that flank the *RAI1* viewpoint (Fig. 2a and b) and which are de facto positive controls, as they were previously reported to interact with *RAI1* in high resolution Hi-C (genome-wide conformation capture) from LCLs [40, 47]. To further corroborate our 4C results we validated selected interactions by 3C-PCR (Additional file 3: Figure S2). Although trans-DNA contacts from Hi-C datasets are only reliable when determined over genomic windows larger than single-restriction fragment [70], we can report consistency between Hi-C results [40] and the interchromosomal and intrachromosomal contacts we identified in this report (Fig. 2c, Additional file 3: Figure S11, Table 1, see “Methods”).

The genes mapping within the *RAI1*-chromatin contacted genomic loci (BRICKs genes) are enriched for genes that encode proteins that interact together (82 observed interactions versus 35 expected; *P* = 6.41e^–12^). BRICKs genes are also enriched for the GO term “detection of light stimulus involved in sensory perception” in Enrichr (*P* = 5.45e^–3^) (see “Methods,” Additional file 2: Table S14). Similarly, Enrichr showed that chromosome contacts were enriched in interchromosomal and intrachromosomal cytobands (17p11, adjusted *P* < 1e^–09^; 17p12, adjusted *P* = 9.7e^–09^; 17p13, adjusted *P* = 1.8e^–03^; and 2q22 adjusted *P* = 4.95e^–02^). *ZEB2*, one of the eight genes found mutated in the SMS individuals, maps to the latter 2q22.3 region and is flanked by BRICKs. To further assess possible functional relationships between *RAI1* and chromatin-contacted genes, we retrieved the list of 322 genes flanking the BRICKs (BRICKs flanking genes, i.e. the closest genes to be found upstream and downstream of a BRICK within a 500 kb window). The 4C assays in particular identified interchromosomal contacts with restriction fragments mapping 200 kb away from the *ZEB2* and *GLDC* gene loci. We then compared the lists of BRICKs genes and BRICKs flanking genes with the list of genes whose expression levels were perturbed in *Rai1* ^–/–^ mouse embryos. Although our analysis is restricted by a small sample size, we found a consistent trend of over-representation (Fisher’s enrichment test, *P* = 0.22, OR = 1.5, and *P* = 0.2, OR = 1.4) with 10 and 18 chromatin-contacted BRICKs genes and BRICKs flanking genes, respectively, differentially expressed in the mouse knockdown model. Interestingly, 6/10 of these BRICKs genes mapping at cytobands 17p13, 17p11 (2 genes), 17q21 and 17q23 (2 genes) have mouse orthologs that map on mouse chromosome MMU11, thus possibly explaining the enrichment of MMU11-mapping genes within genes differentially expressed in *Rai1* ^*–/–*^ mouse embryos (Additional file 3: Figure S12).

## Discussion

Within a cohort of 149 individuals presenting clinical features of SMS we identified 90 % (134/149) of individuals with either a heterozygous deletion of *RAI1* or a predicted deleterious variant of the *RAI1* gene. We used recent advances in genome sequencing technologies to possibly identify genetic alteration(s) associated with SMS in the remaining individuals. These strategies were successfully applied to discover loci associated with ID [71]. They revealed a large genic overlap between ID and ASD, schizophrenia, and epileptic encephalopathy [72], suggesting that some developmental disorders have highly variable clinical presentations. They similarly uncovered limitations to the phenotype-driven strategy and conventional clinical paradigm of identifying individuals with very similar presentations as they revealed an unsuspected phenotypic variance of known disorders [73, 74].

**Fig. 3:**
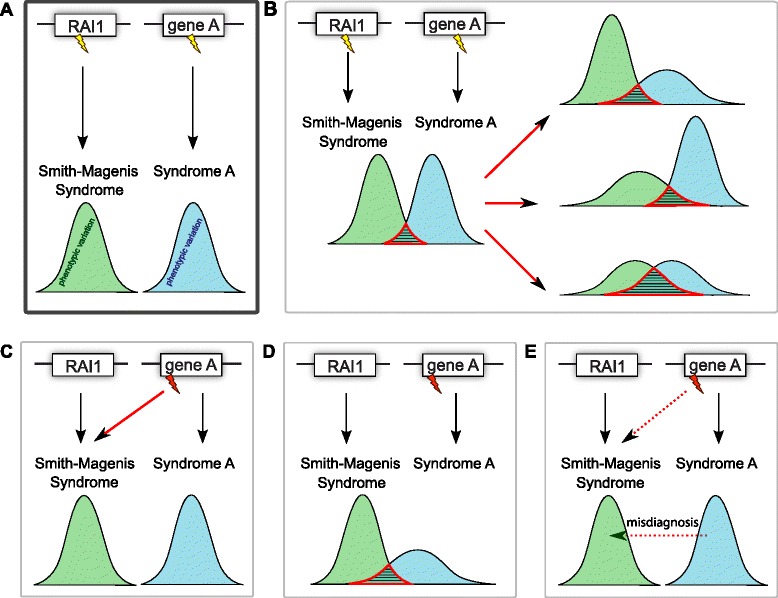
Genetic heterogeneity, phenotypic variance, and misdiagnosis. **a** *RAI1* and gene A are associated with Smith-Magenis syndrome (SMS) and syndrome A, respectively, and variants (*yellow “lightning bolts”*) in those genes cause the green and blue phenotypes, respectively. **b** The phenotypic spectra of these diseases could be more variable than anticipated and result in overlapping features. Such overlap could be due to a broader phenotypic variability of syndrome A, of SMS or of both syndromes (*right panels*). The rare variants in *Rai1*-associated genes identified in individuals with SMS-like features and reported here (*red “lightning bolts”*) could be explained by a combination of genetic heterogeneity of SMS and allelic heterogeneity of gene A (**c**), an increased variance of syndrome A (**d**), or a misdiagnosis of SMS (**e**)

The diagnosis of SMS has primarily relied on clinical suspicion and consideration in a differential diagnosis followed by laboratory studies and confirmatory molecular findings. Since the individuals studied in this cohort were ascertained by experienced clinicians, an aptitude supported by the low number of individuals without a *RAI1* molecular diagnosis, we exploited the remaining 15 individuals to assess the possible heterogeneity of this syndrome (Fig. 3). We identified potentially causal genetic alterations in ten individuals. They comprise variants in the *JAKMIP1*, *ZEB2*, *CASK*, *KMT2D*, *GLDC*, *MECP2*, *MAP2K2*, *KDM5C*, and *POGZ* genes, which are associated with ASD, MOWS, MICPCH, KABUK1, GCE, MRXS13, CFC4, MRXSC, and a new ID syndrome [57], respectively, as well as a 47,XYY karyotype. Interestingly, although 8/15 individuals in this study were male, two of the three mutations on the X chromosome were seen in female patients as opposed to the male patients. Three of the men lack molecular diagnoses at this time, whereas only one woman lacks a credible candidate. Therefore, the over-representation of women with mutations on the X chromosome may be due to small sample size and a lack of recessive X-linked mutations in the cohort or the presence of remaining mutations of interest on the X.

It is important to the medical community to identify phenotypic overlap between diseases, which suggests common causes and alterations of the same pathways, as this knowledge could be exploited therapeutically. In this report, we identify previously unappreciated relationships between SMS and its major driver *RAI1* and other diseases that include MOWS, MICPCH, KABUK1, GCE, MRXS13, CFC4, and MRXSC. Literature mining, co-expression data, transcriptome profiling of *Rai1* ^*–/–*^animal models, and chromosomal contacts support the existence of a comprehensive “biological module” [75] or “disease network” [62] underlying these diseases.

Although none of the 15 individuals described in this study have traditional molecular diagnoses involving *RAI1* haploinsufficiency and thus should formally be considered misdiagnoses, many have phenotypes with considerable overlap with SMS (Fig. 3, Additional file 2: Table S1, Additional file 1: Supplementary text). BAB4947 presented facial dysmorphisms, SMS-like behavioral disturbances that include sleep problems, polyembolokoilamania, onychotillomania, brachycephaly, and brachydactyly, as well as known GLDC-variants associated features such as seizures. His clinical diagnosis could possibly be confounded by the likely presence of two molecular diagnoses: compound heterozygous variants in *GLDC* and an inherited frameshift variant in *TCOF1*, a gene associated with Treacher Collins syndrome-1 (OMIM #154500) and possibly responsible for the down-slanting eyes, everted lateral eyelids, and malar hypoplasia. The clinical scenario is similar with cases BAB2474 and BAB2540, who did not show CFC4- (e.g. ectodermal anomalies, craniofacial features) and MICPCH-distinctive features (e.g. microcephaly and pontocerebellar hypoplasia). Likewise individual BA2492 has a constellation of symptoms (sleep disturbance, DD, cognitive impairment, brachydactyly) compatible with only the most severe 47,XYY sex chromosome aneuploidy cases [76]. Consistent with the hypothesis of expanded phenotypes, the phenotypic variability of White-Sutton syndrome associated with variants in *POGZ* keeps extending with clinical features including ASD, DD, ID, schizophrenia, and microcephaly [57, 71, 77–83]. We can also not formally rule out that we have not yet determined the true genetic cause(s) of the phenotypic spectrum of these individuals or they occur in presence of more complex, blended phenotypes as exemplified by BAB4947 above. BAB2451 harbors a “probably pathogenic” variant (SIFT HumDiv score = 1; HumVar score = 0.982) in the gene *JAKMIP1*. Recent findings have linked the loss of *JAKMIP1* to neuronal translation dysregulation during synaptic development; mice knocked out for the *JAKMIP1* paralog display social deficits, stereotyped activity, altered vocal communication, increased impulsivity, and other autistic-like behaviors [84].

The presented results support the notion that at least some of the identified variants in candidate SMS contributory genes *CASK*, *GLDC*, *KDM5C*, *KMT2D*, *MAP2K2*, *MECP2*, *POGZ*, and *ZEB2* are causative of the observed phenotypes and thus that modification of the function of these genes is associated with a greater phenotypic variability than previously expected (Fig. 3). Conversely, one and two carriers of damaging *RAI1* variants were identified within a total of 6381 ASD [79, 85] and 2426 ID [71, 78, 86–89] individuals, respectively. Whereas the phenotype of one of the ID individuals was retrospectively found to be consistent with SMS [89], we lack detailed phenotypic information regarding the other two cases. If we assume that these two individuals do not present with typical SMS features that would have excluded them from these cohorts, it suggests that the phenotype of carriers of *RAI1* deleterious variants is similarly more variable than anticipated.

Structural variations, especially large rearrangements involving several genes, shape tissue transcriptomes and impact the expression of genes mapping to their flanks [64, 90]. We show that the homozygous deletion of *Rai1* in mouse embryos [32] influences the expression of several genes and in particular MMU11 genes. Furthermore, the *RAI1* viewpoint contacts the orthologous genes at the chromatin level. As some of these genes contribute to phenotypes associated with *RAI1* variation (e.g. *KRT17* with “hoarse voice” (HP:0001609), *B9D1* with “low-set, posteriorly rotated ears” (HP:0000368), “hypertelorism” (HP:0000316), and “microcornea” (HP:0000482)), they could be involved in *RAI1* pathways. The relevance of using 3C-based approaches as unbiased tools to discover clinically related genes is reinforced by their successful application in assessing connected regions involved in similar phenotypes [35] and genes interacting with risk loci identified in genome-wide association studies (GWAS) [91, 92]. The contacted regions encompass candidate genes involved in “detection of light stimulus” and related gene ontology terms. These processes all refer to photodetection, which controls circadian rhythm and melatonin production from the pineal gland. RAI1 is an important player in this mechanism, by controlling the transcriptional levels of CLOCK, a key component of the mammalian circadian oscillator that transcriptionally regulates many critical circadian genes [93]. Another gene mapping within the SMS critical region on chromosome 17p11.2 and linked to these processes is the subunit 3 of the COP9 signal transduction complex (COPS3), essential for the light control of gene expression [94]. It is thus possible that the disruption of the orthologous locus in the *Rai1* ^*–/–*^ mice perturbs chromatin loops and affects expression levels of *RAI1*-contacted/functionally associated genes. We are well aware of the limitations of using LCLs in this type of study, and particularly to assess chromatin contacts between genes whose expression specificity resides in other cell lineages. These experiments are nevertheless worth pursuing simply because: (1) the primary human target tissues often remain beyond reach; (2) we cannot exclude a broad to ubiquitous expression pattern for the genes involved in these disease processes; and (3) long range chromatin contacts were shown to be stable across cell lines and tissues regardless of expression status [95]. Similar limitations apply to the use of embryonic stem cell-derived material, while animal tissues have a different set of shortcomings.

## Conclusions

Our results strongly support a disease network associated with *RAI1* and illustrate the utility of a comprehensive multifaceted diagnostic approach even in the presence of a distinctive disorder.

## Additional files

Additional file 1: Supplementary text. (DOCX 121 kb)
Additional file 2: Fourteen supporting tables. A table caption of each is given within the file. (XLSX 1540 kb)
Additional file 3: Twelve supporting figures. A figure caption for each is given within the file. (PDF 33990 kb)
