# Supplementary material for: Identification of a RAI1-associated disease network through integration of exome sequencing, transcriptomics, and 3D genomics
Source: Genome Med. 2016 Nov 1;8:105. doi: 10.1186/s13073-016-0359-z (PMC5088687; doi:10.1186/s13073-016-0359-z)
Supplement: Additional file 1: — Supplementary text. (DOCX 121 kb) [file 13073_2016_359_MOESM1_ESM.docx]

**Additional file 1**

**Supplementary Text**

*Detailed SMS patients’ phenotypes*

BAB1604 was a 3-years-2-months old female with global developmental delay, behavioral difficulties, self-injurious behavior, and stereotypies including hand flapping and self-hugging. Sleep cycle was reportedly normal. The patient was reported to have always been hypotonic, and had a femur fracture at 11 months with an otherwise unremarkable skeletal survey. Family history was noncontributory. Growth parameters showed a head circumference at about 5th percentile despite height and weight around the 75th percentile. Features were somewhat coarse with deep-set eyes and synophrys, a small philtrum, thick lips with a cupid's bow, wide spaced teeth, a prominent chin and borderline low set ears. She had hypotonia, joint hypermobility, bilateral muscle wasting of her distal thighs and calves, and absent deep tendon reflexes (DTRs) in the lower extremities. Toenails were hypoplastic and she had bilateral 5th toe clinodactyly. Previous work-up included urine organic acids, plasma amino acids, oligosaccharides, mucopolysaccharides, very long chain fatty acids, ceruloplasmin, liver function tests, B12, folate, lactate, TSH, lead level, creatine kinase, high resolution chromosomes, and FISH for deletion in the Prader-Willi region - all normal. Ophthalmology exam was normal. Brain MRI demonstrated an asymmetric cranial vault but was otherwise normal. EMG demonstrated the possibility of an axonal neuropathy consistent with Charcot-Marie-Tooth disease.

BAB1952 was a male evaluated at 13-years for global developmental delay, behavioral difficulties including aggressive outbursts and self-injurious behavior, nail biting, and sleep disturbances. He was reported to have had feeding difficulties as an infant, hypotonia, and a severe articulation disorder. MRI showed a mild increase in the CSF spaces around the cerebral hemispheres. Renal ultrasound demonstrated bilateral hydronephrosis with hydroureters, and bilateral ureteropelvic junction obstruction status post surgical correction.

BAB2134 was an 11-year old male with developmental delay, aggressive behavior and bilateral sensorineural hearing loss. He was born at 38 wks of gestation, and weighed 6 lbs 8 oz (appropriate for gestational age, AGA). The patient had slightly delayed motor development, with walking at 18 months. Speech and language development were also delayed, with first words spoken at 4 years. He was reported to have an abnormal sleep pattern, self-injurious behavior including pulling out his toenails, and aggression towards others. His head circumference was at the 40th percentile, height was 50th percentile, and weight was 90^th^ percentile. Physical exam revealed frontal bossing, chubby cheeks, micrognathia, upslanting palpebral fissures, a flat bridge nose with scooped out root, and brachydactyly and clinodactyly of the 5th finger bilaterally.

BAB2293 was a male evaluated at 2-years-3-months for global developmental delay and behavioral difficulties. He was born by emergency C-section for failure to progress secondary to deflexed brow presentation. Birth weight was AGA; head circumference at birth was just above the 90^th^ percentile. He had delayed motor development, with commando crawling at 18 mo, and was not yet walking when evaluated. Speech and language development were delayed, with a single word "mum" at 27 months. He exhibited aggressive behaviors, hand flapping, and self-injurious behaviors including biting his hands and toes, head banging, and pulling out his own hair. He had an abnormal sleep pattern and chronic constipation. Head CT was unremarkable aside from deformational plagiocephaly and brachycephaly. Work-up included chromosome analysis, Fragile X testing, thyroid function studies and a urine metabolic screen, all of which resulted normal. Whole exome sequencing showed a variant in *KDM5C* that is associated with X-linked syndromic mental retardation Claes-Jensen type (OMIM#300534).

BAB2321 was a 12-year old male with developmental delay, ADHD, obsessive-compulsive disorder, and aggressive behavior. Height, weight and head circumference were symmetric, at the 5^th^-10^th^ percentiles. The patient had an overall coarse appearance, and dysmorphic features included a narrow head, keel-shaped forehead, low anterior hair line, deep-set eyes, slight periorbital fullness, epicanthal folds, a wide mouth with everted tented upper lip, wide spaced teeth with malocclusion, macroglossia, slightly high-arched palate, marked prognathism, and prominent ears. Hands and feet were short with stubby digits, and prominent fingertip pads. Skin involvement included areas of psoriasis. Previous work-up including chromosome analysis, telomeres, Fragile X testing, and enzyme assays for lysosomal storage disorders resulted normal.

BAB2330 was a 5-years-8-months old female evaluated for acquired microcephaly, speech and language delay, and behavioral abnormalities including self-injurious behaviors. She had a disturbed sleep cycle that responded to melatonin. During infancy, seizure-like episodes were witnessed but EEG and brain imaging were normal. The patient was born at full term after an uncomplicated pregnancy. Birth weight was 6 lbs 10 oz (AGA). Weight and length during infancy were around the 5th percentile, but weight had increased to 97th percentile by age 5-years-8-months. Length at this time was 25^th^ percentile. Head circumference, which had been microcephalic during toddler years (-4SD), was at 5^th^-10^th^ percentile at 5.5 years. Physical exam showed prominent forehead, narrow eyes with mildly upslanting palpebral fissures, small nose, and somewhat thin upper lip. Brain MRI and EEG were normal, as were ophthalmology evaluation, chromosome analysis, FISH for 17p11.2 and 22q11.2 deletions, lead levels, thyroid function studies, plasma amino acids and urine organic acids. Whole exome sequencing showed a *de novo* variant in *POGZ*[1].

BAB2451 was a 10-year old female with developmental delay, intellectual disability, seizures, self-injurious behavior, obesity, onychotillomania, and narcolepsy. Physical examination showed macrocephaly and a down-turned mouth; otherwise, the patient was non-dysmorphic. MRI showed mild global volume loss.

BAB2474 was a 5-year old female with developmental delay, feeding problems and microcephaly. She was born at 33 wks gestation by C-section due to premature onset of labor, acute development of oligohydramnios and breech presentation. Birth weight was 4 lbs 1 oz (50^th^ percentile), and Apgars were 9 and 9 at 1 and 5 minutes. The patient had difficulty feeding and failure to thrive, necessitating G-tube feeds for the first four years of life. She had motor delay, with walking at 3.5 years; and speech and language delay. She had seizure-like activity but a normal EEG and MRI. Ophthalmology diagnosed ocular motor apraxia, and she needed eyeglasses since age two years. She had bilateral externally rotated legs and flexible valgus deformity in the feet. She had new onset of hypertension at 5 yrs, with normal renal parenchyma per ultrasound; however, renal arteries were not visualized and follow up imaging was recommended. Previous work up included chromosome analysis, methylation studies for Angelman syndrome, and FISH for 17p11.2, 22q11.2, 15q11, 15q13 and 4p deletions - all negative. Whole exome sequencing showed a variant in *MAP2K2* that is associated with cardiofaciocutaneous syndrome (OMIM #615280).

BAB2492 was a 3-year old male with developmental delay, intellectual disability, hypotonia, seizure-like activity but normal EEG, feeding difficulties, failure to thrive and brachydactyly. Chromosome analysis showed 47,XYY.

BAB2540 was born after a normal pregnancy, at a birth weight of 4350 grams. She walked at 18 months. Speech development was severely delayed. She exhibited aggressive behavior, self-injury, placing objects in orifices (nose and ears), and hand biting. She had amblyopia and normal hearing. Coarse facial features were apparent, and she had large hands. MRI was reportedly normal, as were chromosome analysis and FISH for 17p11.2 deletion. Whole exome sequencing showed a *de novo* variant in *CASK*, which is associated with mental retardation and microcephaly with pontine and cerebellar hypoplasia (OMIM #300749).

BAB2552 was a female evaluated at 5 years for developmental delay, a very short attention span and behavioral problems. She had an abnormal sleep pattern and self-injurious behaviors including self-biting. Physical exam showed deep-set eyes, and overall craniofacial features were felt to be consistent with Smith-Magenis syndrome (no details available). Whole exome sequencing showed a variant in *MECP2*, which is associated with MECP2 X-linked syndromic mental retardation 13 (OMIM #300055).

BAB2559 was an 8-year-old male evaluated for developmental delay and behavioral difficulties. He exhibited speech delay, aggressive behavior, ADHD, bipolar disorder, self-injurious behavior, and sleep disturbances. He was reportedly hypotonic during infancy, and had difficulty running at 8 years. Physical exam revealed poor dental alignment, obesity specifically in the lower abdomen and pubic area, and absent DTRs in the lower extremities. MRI showed mild widening of CSF spaces around the cerebral and cerebellar hemispheres and mild thinning of the corpus callosum. Previous evaluation was negative, including FISH for 22q11.2 deletion and telomere FISH.

BAB4947 was a male patient born to consanguineous parents of Turkish descent, with intellectual disability, seizures, behavioral disturbances, aggressive behavior, polyembolokoilamania, and onychotillomania. Physical appearance was striking for a Kabuki-like face. He had brachycephaly, arched eyebrows, long palpebral fissures with lateral eversion of the eyelids, a thick and everted upper lip, high arched palate, ear lobe hyperplasia, bilateral preauricular pits, bilateral single palmar creases, and brachydactyly. Chromosome analysis and FISH for 17p11.2 resulted normal. Whole exome sequencing showed compound heterozygote variants in *GLDC* and an inherited frameshift variant in *TCOF1,* which are associated with glycine encephalopathy (OMIM #605899) and Treacher Collins syndrome-1 (OMIM #154500), respectively.

**References**

1. White J, Beck CR, Harel T, Posey JE, Jhangiani SN, Tang S, Farwell KD, Powis Z, Mendelsohn NJ, Baker JA *et al*: **POGZ truncating alleles cause syndromic intellectual disability**. *Genome medicine* 2016, **8**(1):3.
