# Supplementary material for: Identification of a RAI1-associated disease network through integration of exome sequencing, transcriptomics, and 3D genomics
Source: Genome Med. 2016 Nov 1;8:105. doi: 10.1186/s13073-016-0359-z (PMC5088687; doi:10.1186/s13073-016-0359-z)
Supplement: Additional file 3: — Twelve supporting figures. A figure caption for each is given within the file. (PDF 33990 kb) [file 13073_2016_359_MOESM3_ESM.pdf]

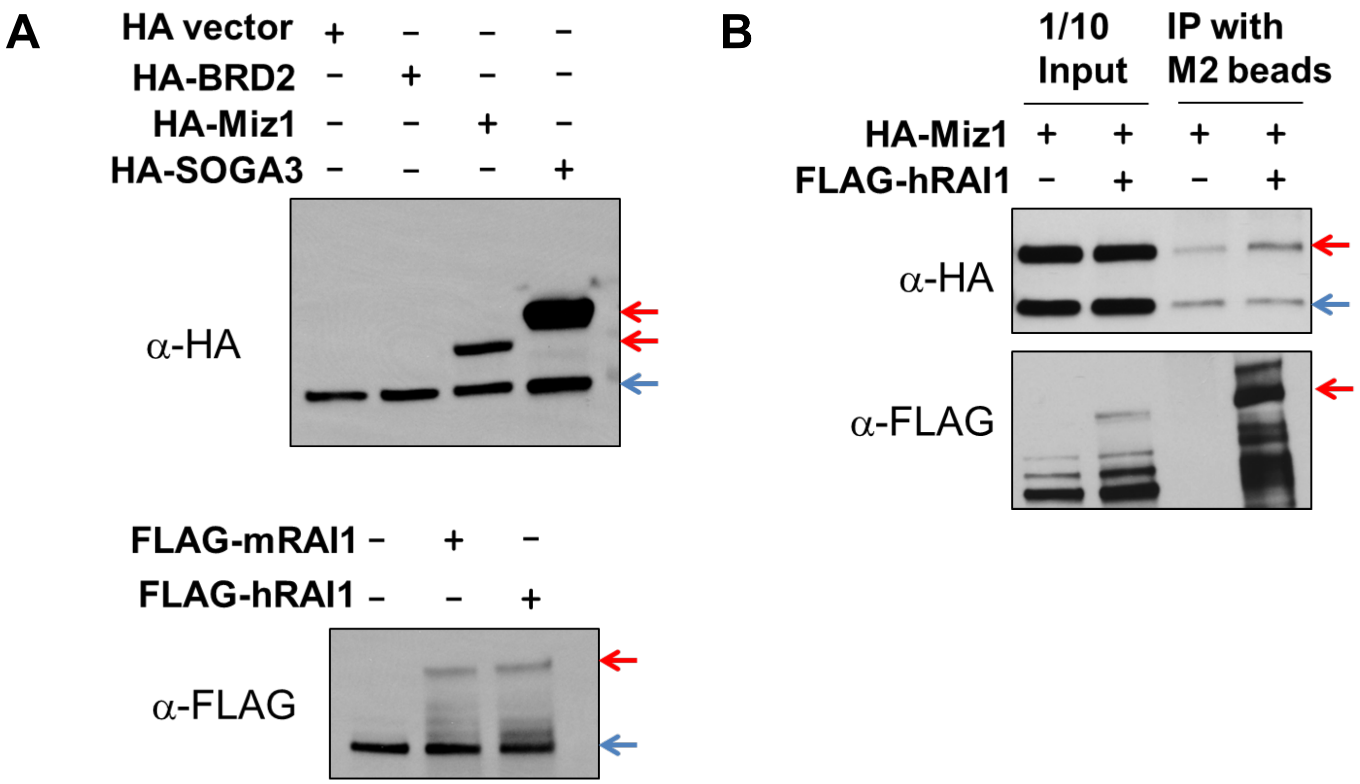

Figure S1: Confirmation of yeast two-hybrid data A. Testing the expression of BRD2, MIZ1, SOGA3, RAI1 expression vectors. Red arrow: the expected products. Blue arrow: non-specific bands. BRD2 does not express at all and other tagging strategies were used but still did not express (data not shown). MIZ1 construct has mild expression while the SOGA3 construct displays strong expression. B. RAI1 interacts with MIZ1 in the Co-IP experiment. Red arrow: the expected products. Blue arrow: non-specific bands.

A

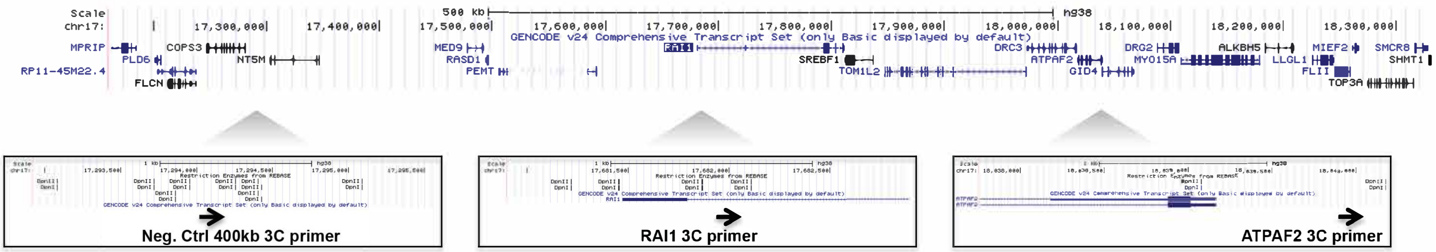

B

RAI1 3C primer-ATPAF2 3C primer

1. **GRAEM 3C (0.625µl-1.25µl-2.5µl)**
2. **SOMAG 3C (0.625µl-1.25µl-2.5µl)**
3. **GRAEM 3C/-Ligase (0.625µl-1.25µl-2.5µl)**
4. **SOMAG 3C/-Ligase (0.625µl-1.25µl-2.5µl)**
5. **dH_2O**

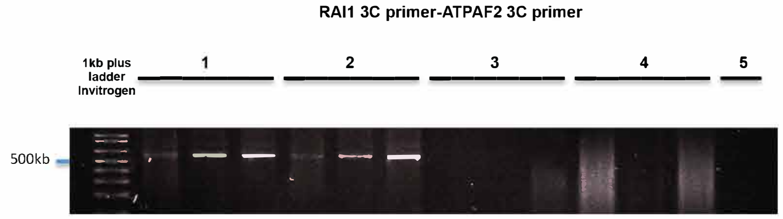

RAI1 3C primer-Neg. Ctrl (400kb) 3C primer

6. **GRAEM 3C (2.5µl)**
7. **SOMAG 3C (2.5µl)**
8. **GRAEM 3C/-Ligase (2.5µl)**
9. **SOMAG 3C/-Ligase (2.5µl)**
10. **dH_2O**

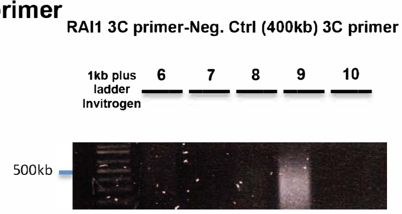

Oligo Name	Sequence 5' to 3'
RAI1_3C_DpnII	GTCCTTGCTTTGTGTGGTGTG
ATPAF2_3C_DpnII	AGAAGAGCAAGGGGAACTCGTA
NegativeCtrl_400kb_3C_DpnII	CCAGAGTATGGCCTGTCTTTTT

C

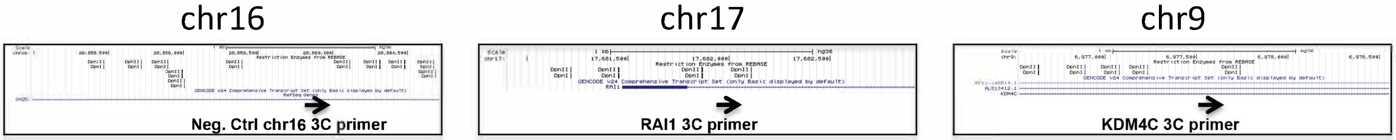

D

RAI1 3C primer- KDM4C 3C primer

1. **GRAEM 3C (0.625µl-1.25µl-2.5µl)**
2. **SOMAG 3C (0.625µl-1.25µl-2.5µl)**
3. **GRAEM 3C/-Ligase (0.625µl-1.25µl-2.5µl)**
4. **SOMAG 3C/-Ligase (0.625µl-1.25µl-2.5µl)**
5. **dH_2O**

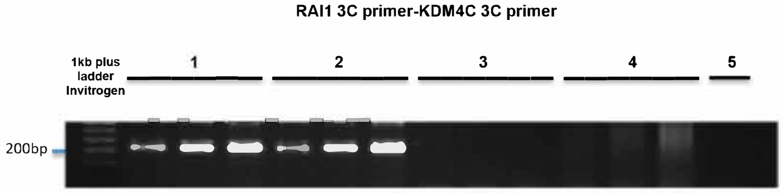

RAI1 3C primer-Neg. Ctrl (chr16) 3C primer

6. **GRAEM 3C (2.5µl)**
7. **SOMAG 3C (2.5µl)**
8. **GRAEM 3C/-Ligase (2.5µl)**
9. **SOMAG 3C/-Ligase (2.5µl)**
10. **dH_2O**

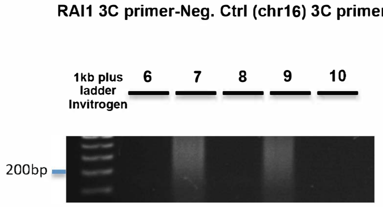

Oligo Name	Sequence 5' to 3'
RAI1_3C_DpnII	GTCCTTGCTTTGTGTGGTGTG
KDM4C_3C_DpnII	CCTCCAGTTTCAGTTGTTTGCT
NegativeCtrl_chr16_3C_DpnII	ATAACGTCCCCACCAACCTT

Figure S2: Chromatin loops validation
(A) UCSC genome browser tracks of chr17p11.2 (approximately +/- 500kb from RAI1, GRCh38/hg38 assembly) and zoom-in (black rectangles) of DpnII restriction sites and 3C primers location (black arrows), selected at RAI1, ATPAF2 loci and control equidistant loci.
(B) 3C-PCR validation of 4C and Hi-C-detected RAI1-ATPAF2 interaction (see Figure 2), using control 3C templates (GRAEM and SOMAG, with titration) and unligated 3C templates (GRAEM and SOMAG/-Ligase, with titration) (upper panel). The expected specific PCR band is only detected in 3C ligated templates when using RAI1-ATPAF2 3C primers, while no specific band is visible when RAI1 3C primer is tested with 3C primer designed at the equidistant control region (Neg. Ctrl 400kb, lower panel). Primer sequences available in the figure table.
(C) Zoom-in UCSC genome browser tracks at RAI1 (chr17p11.2), KDM4C (GLDC flanking gene, chr9p24.1) and control locus at 16p11.2, showing locations of DpnII restriction sites and 3C primers (black arrows).
(D) 3C-PCR validation of 4C-detected RAI1-KDM4C interaction (see Table S11), using control 3C templates (GRAEM and SOMAG, with titration) and unligated 3C templates (GRAEM and SOMAG/-Ligase, with titration) (upper panel). The expected specific PCR band is only detected in 3C ligated templates when using RAI1-KDM4C 3C primers, while no specific band is visible when RAI1 3C primer is tested with 3C primer designed at 16p11.2control region (Neg. Ctrl chr16, lower panel). Primer sequences available in the figure table.

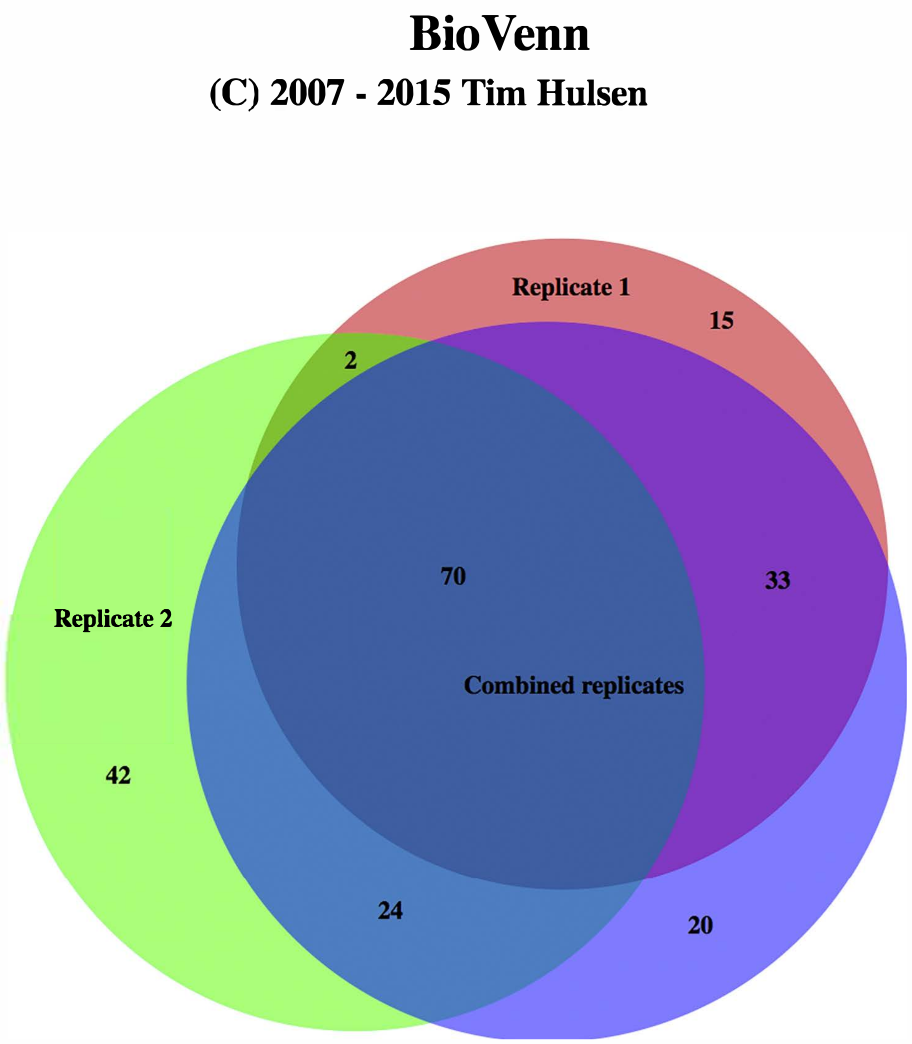

Figure S3: Comparison of 4C-seq replicates
Venn diagram showing the overlap between the BRICKS obtained after averaging replicates (combRep) compared to BRICKS found in each replicate separately (rep1 and rep2).

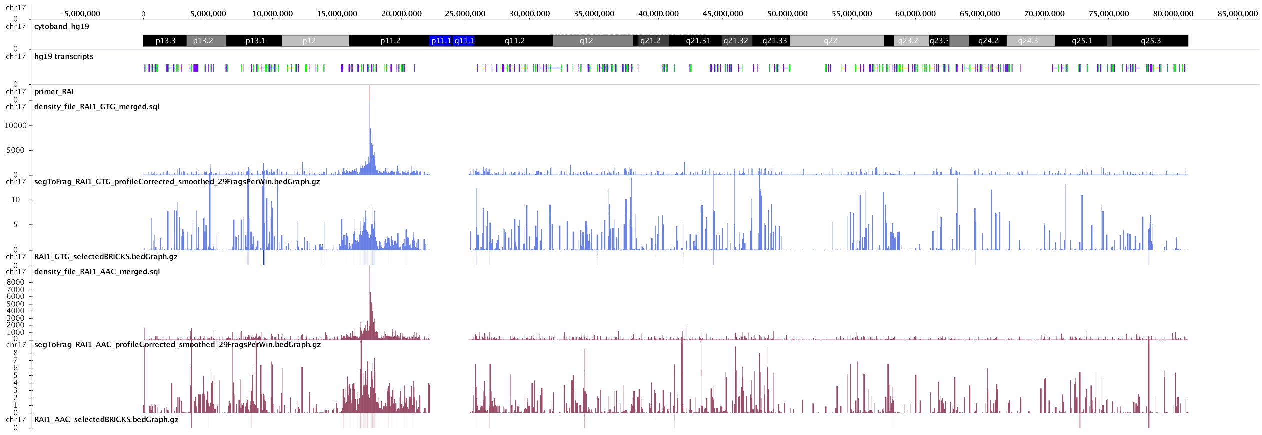

Figure S4: Local 4C interactions
Local 4C interactions profile of RAI1 (Panels from top to bottom)
Hg19 cytoband and transcripts: Human chromosome 17 cytobands and structure of the transcripts along the whole chromosome.
Viewpoint: The red tick shows the mapping position of the RAI1 viewpoint used in the 4C experiments.
Density/PC/BRICKs of replicates 1 and 2: Density (upper part of each panel), smoothed and profile-corrected 4C signal (middle part) and BRICKs (lower part) identified for each replicate (blue and burgundy, respectively).

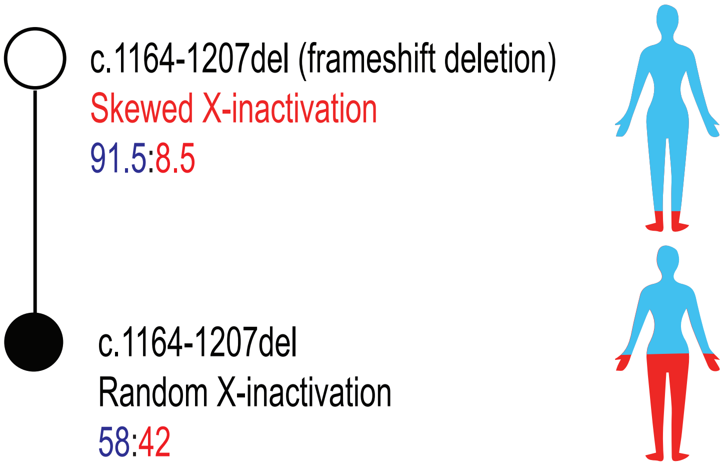

Maternally inherited *MECP2* frameshift deletion. X-inactivation studies revealed highly skewed inactivation in the mother against the variant but not in the affected daughter.

Figure S5: X-inactivation in patient BAB2552 and her mother
X-inactivation analyses of patient BAB2552 (top) and her mother (bottom).

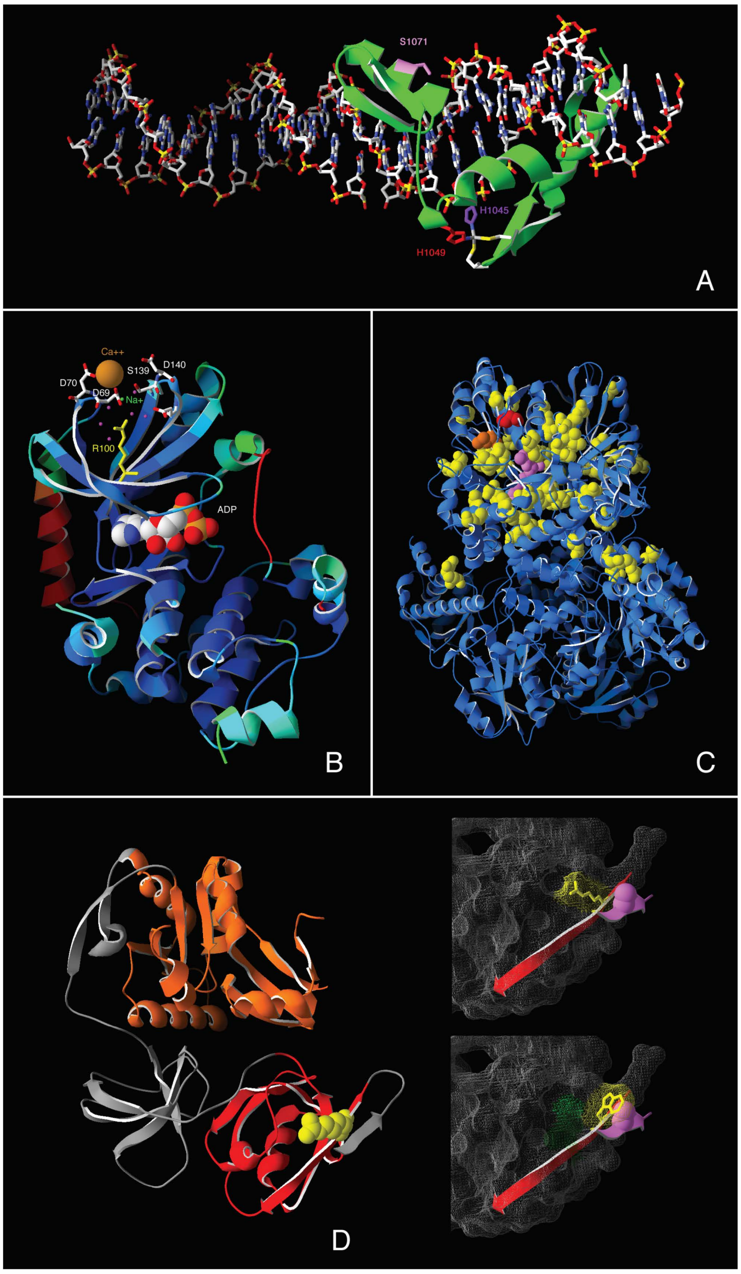

Figure S6: Modeling of variants identified in SMS patients

(A) ZEB2: Position of the H1049P variant identified in patient BAB2386 with respect to the Zinc finger residues 995-1078 (green ribbon). Residue H1049 (red) together with residue H1045 (purple), which was reported mutated in a Mowat-Wilson syndrome patient (Supplementary Table S10), is essential for the coordination of the Zn++ ion of the Zinc finger. The S1071 residue located within an alpha-helix that directly contacts the DNA major groove (pink) was similarly found mutated in another MOWS patient (Supplementary Table S10). A proline at this position will likely disrupt the helix through steric hindrances, thus impacting the DNA binding ability of the Zinc finger.

(B) MAP2K2: Position of the D69del variant identified in patient BAB2474 with respect to the overall MAP2K2/MAP2K1 structures. Since (i) MAP2K1 has a higher resolution than MAP2K2; (ii) is readily superposable onto MAP2K2; and (iii) residues close to D69del in 3D space are strictly conserved, its model has been used to highlight the context of the variant. The ribbon representation is colored by backbone root-mean-square deviation to MAP2K1 (with a dark blue, blue, cyan, green gradient representing region with rmsd < 0.5, < 1.0, < 1.5, < 2.0 respectively). Regions not resolved in MAP2K2 are shown in red and ADP is represented as a space-filling model. The D69 and D70 residues are located at the top of a conserved GELKDD loop facing the conserved residues S139 and D140. These residues are involved in the binding of a Ca++ ion (orange). D69 is also binding a Na+ ion (green), itself coordinated by a network of water molecules (purple) held in place by the conserved R100 (yellow) and E138 (not highlighted). A deletion of D69 will alter the local loop geometry and disrupt the ion coordination ability of this conserved region.

(C) GLDC: Positions of the compound heterozygote variants P647L (red space-filling model) and L726Q (orange space-filling model) found in patient BAB4947 relative to the 61 missense variants conferring glycine encephalopathy (GCE) reported in the literature, i.e. R59T, L82W, S132W, Y138F, N150T, Y161C, G171A, T187K, A202V, R212K, C225R, T269M, A283P, D295Y, L296R, A313P, P329T, H371D, I372F, R373W, A389V, R410K, I440N, R461Q, P509A, R515S, T532R, N533Y, R536E, M552V, S564I, A569T, E597K, V611G, C644F, H651R, A694P, P700A, S701F, V705M, G728R, N732K, R739H, H753P, H760Q, G761R, G762R, P765S, G766C, P769L, G771R, H775R, R790W, A802V, T830M, M840K, A841P, V905G, P949L, S957P and R966G (yellow space-filling models). Although disease-conferring variants are scattered throughout the peptide chain, once analyzed in their 3D context they cluster in a specific structural region, which in its center bears the catalytic lysine K754 and pyridoxal phosphate (pink space-filling model). All GCE-conferring variants result in bulkier residues or elicit a change of charge (Supplementary Table S7). The variants identified in the SMS patient cannot, likewise, be accommodated without forcing a local structural rearrangement; specifically P647L clashes with I676, whereas L726Q collides with P740 and Q734.

(D) CASK: Two partial CASK crystal structures covering residues 487-572 (red) and 739-914 (orange) were superposed onto the crystal structure of PALS1/Crb to model their relative orientation and highlight the position of the R489 residue (yellow space-filling model) found mutated in patient BAB2540 in context of the overall C-terminal domain of the protein (left panel). The R489 residue (yellow space-filling model) adopts an extended conformation perpendicular to the beta sheet (red; top right panel). Whereas in some species a Lysine residue is found at this position as it can adopt a similar conformation and be positively charged, the mutant R489W identified in SMS patient BAB2540 cannot be accommodated in this structural context. A bulkier tryptophan residue in this position will have to point outward in a direction parallel to the beta-sheet (bottom right panel). Since it is located at the end of a domain, the protrusion of an unexpected sidechain in this region could prevent the formation of an extended beta-sheet, for example with the N-terminal domain of CASK or another protein, because of collision with its sidechains. The position of a beta-strand present in the crystal structure of PALS1/Crb (pink arrow) is shown to illustrate this concept with R489 (top right panel) and W489 (bottom right panel). The R489W variant will affect the molecular surface of the domain, revealing a recessed hydrophobic patch formed by the hydrophobic residues V491 (dark green) and I567 (light green), which possibly will affect interaction with other molecules.

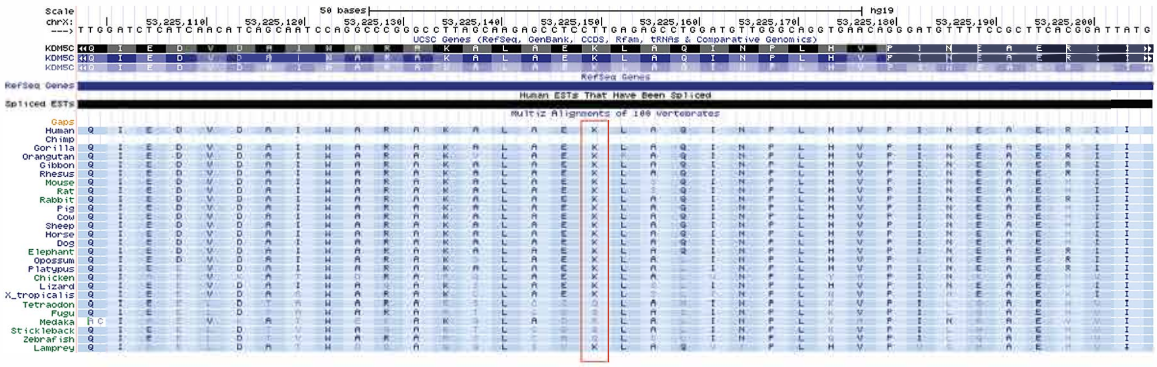

Figure S7: Conservation of the K1023 residue of KDM5C
PhyloP and Multiz Alignment UCSC tools showing the conservation of the K1023 residue of KDM5C (position highlighted by a red rectangle) across vertebrate species (human, chimp, gorilla, orangutan, gibbon, rhesus, mouse, rat, rabbit, pig, cow, sheep, horse, dog, elephant, opossum, platypus, chicken, lizard, xenopus tropicalis, tetraodon, fugu, medaka, stickleback, zebrafish and lamprey).

all tissues

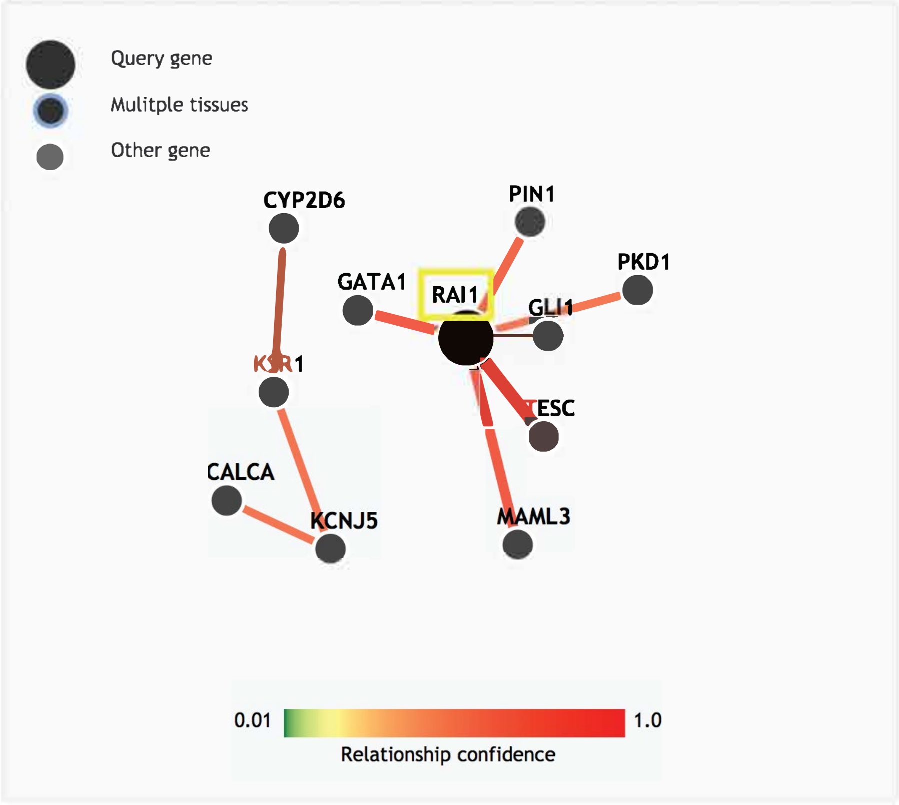

neuron

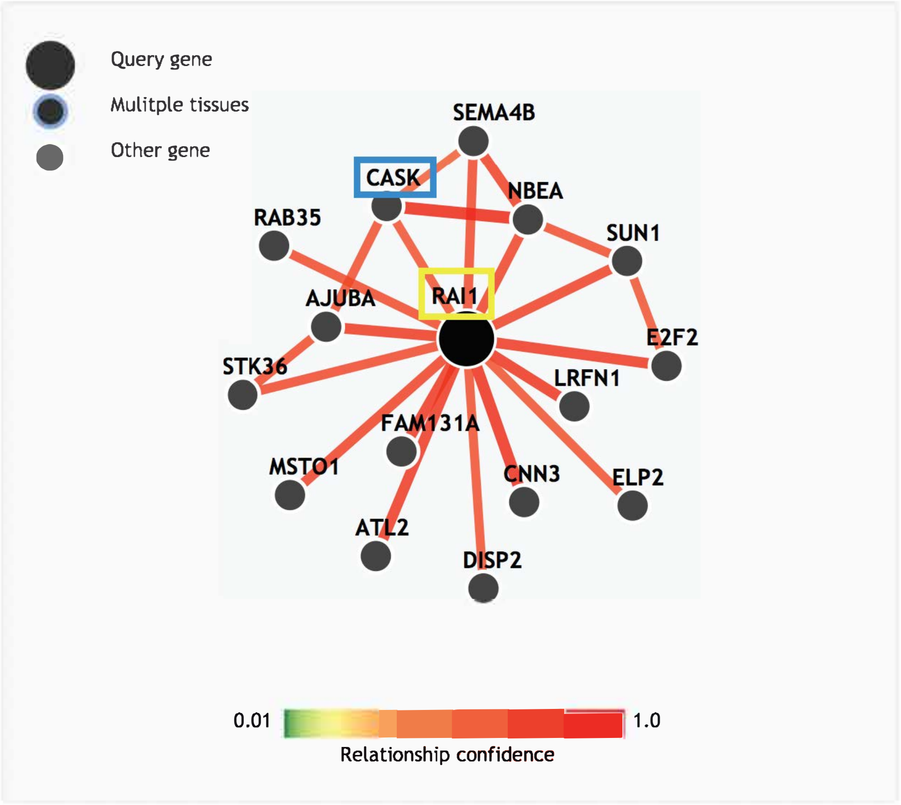

Figure S8: Co-expression-based molecular interactions
Tissue-specific functional interaction network built using GIANT (Genome-scale Integrated Analysis of gene Networks in Tissues, http://giant.princeton.edu/) in "all tissues" (top panel) versus "neuron" (bottom panel), with minimum relationship confidence = 0.5 and maximum number of genes = 15. RAI1 was used as query (relationship confidence: 0.58; 77% due to co-expression).

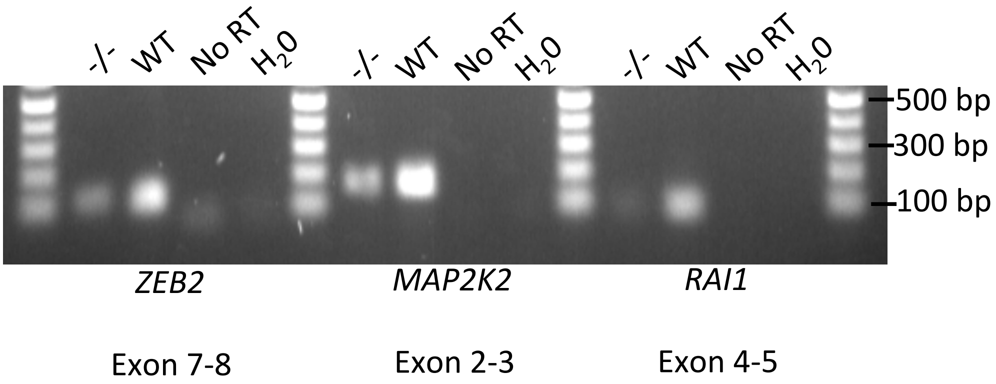

Figure S9: Expression level validation
To confirm microarray analyses on Rai1-/- embryos suggestive of lower Zeb2 and Map2k2 expression, semi-quantitative RT-PCR was conducted on three separate RT reactions from a Rai1-/- embryo (-/-) and a wild-type control littermate embryo (WT) (representative results depicted). PCR was conducted using primers designed within exonic regions to detect Zeb2, Map2k2, and Rai1 transcripts (see Methods section for primer sequences)

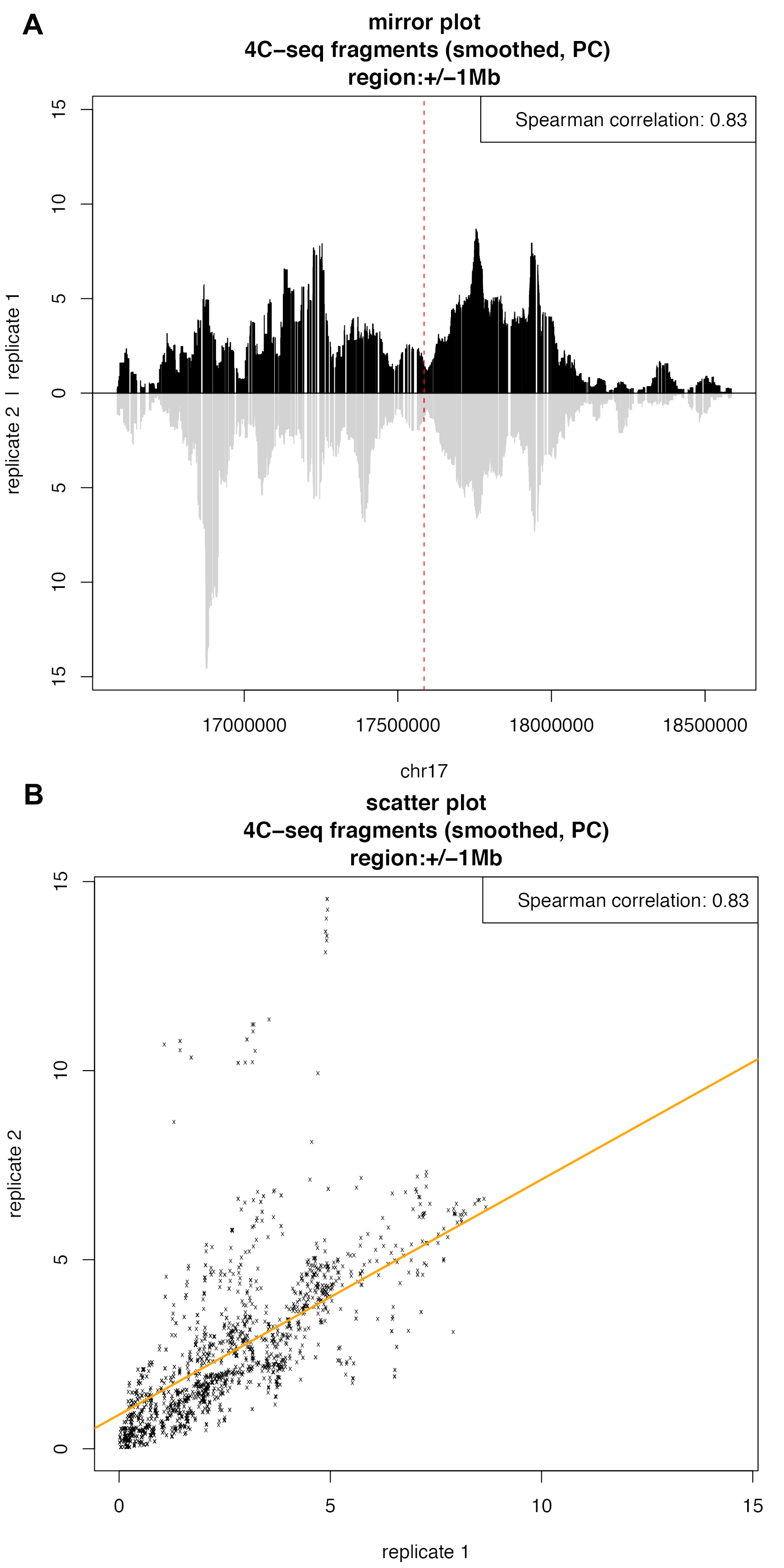

Figure S10: Comparison of 4C-seq replicates
Mirror (A) and Scatter plot (B) of 4C-seq profile of replicate 1 (top portion) and 2 (bottom portion) in the region defined as viewpoint +/- 1Mb with Spearman's rank correlation calculated between the two replicates.

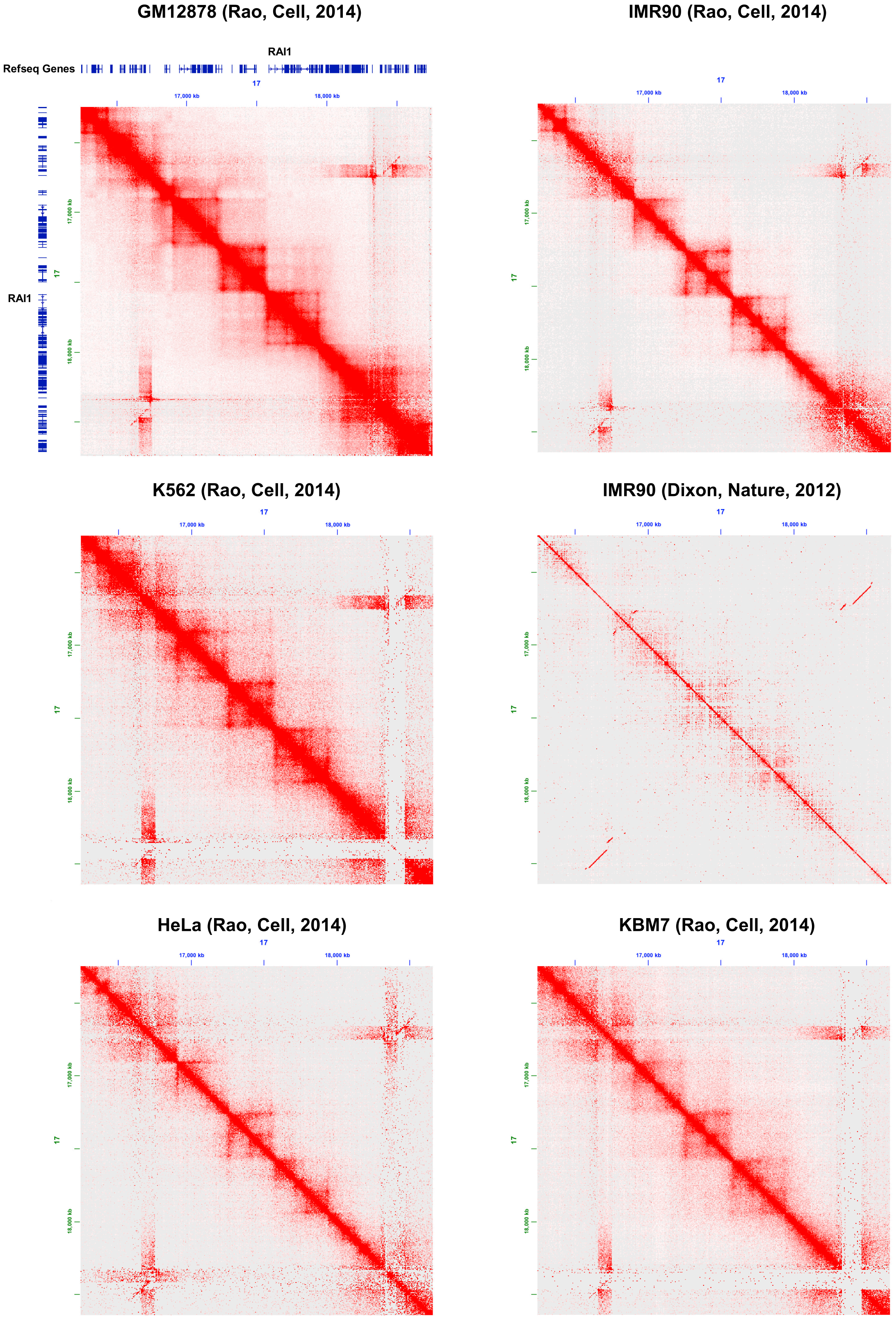

Figure S11: High resolution Hi-C chromosome conformation capture results obtained with the GM12878 LCL within the chromosome 17 17.25-18.75Mb window (5kb resolution, coverage normalization) confronted with different cell lines as IMR90, K562, HeLa and KBM7[47, 48], visualized by Juicebox (http://www.aidenlab.org/juicebox/). The position of the RAI1 gene is indicated.

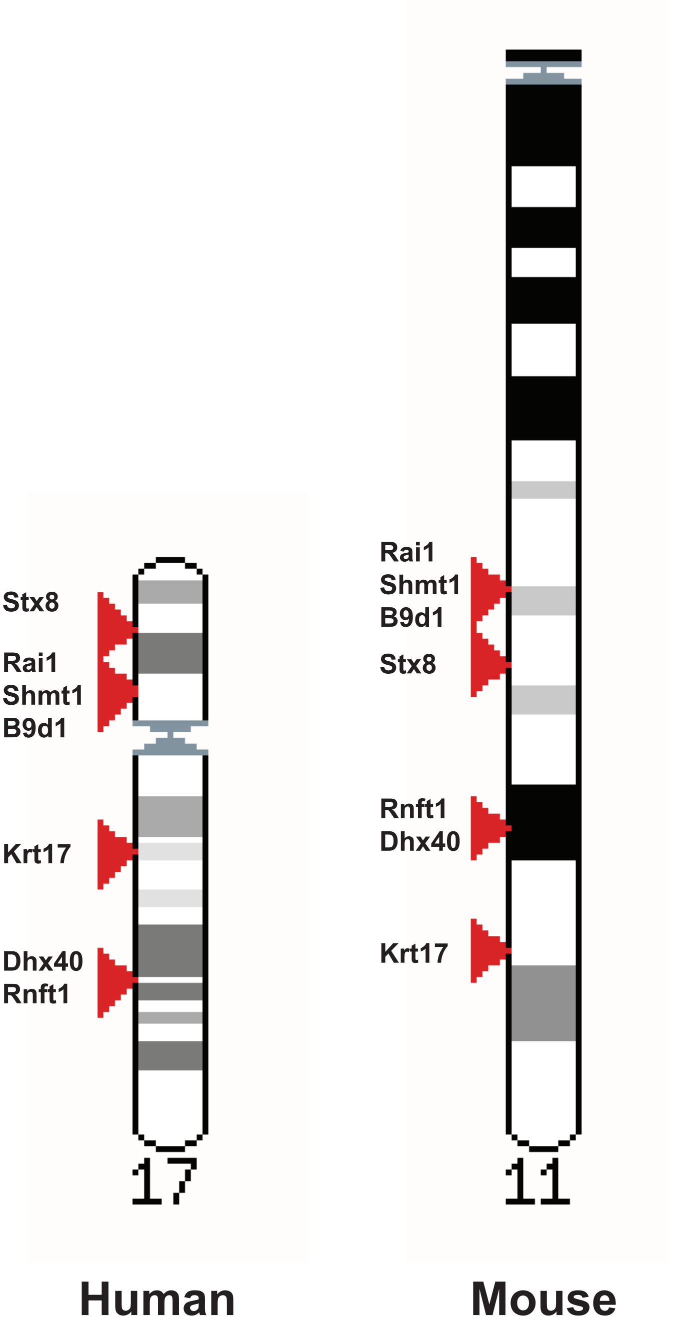

Figure S12: Mapping of the HSA17 BRICKs genes and their mouse MMU11 orthologs differentially expressed in 10.5 dpc Rai1-/- mouse embryos.
